# Pharmacological Targeting of Type H Endothelial Cells in Knee Osteoarthritis: From Molecular Signaling to Cellular Homeostasis

**DOI:** 10.3390/cells15141312

**Published:** 2026-07-22

**Authors:** Chunlu Yan, Chuangwei Sui, Qiao Wan, Xupeng Liu, Zeling Fang, Jiarong Shi, Chen Chen, Yu Jiang, Juan Yu, Fangyu An

**Affiliations:** 1 Dunhuang Medical Academy, Gansu University of Chinese Medicine, Lanzhou 730000, China; 2 School of Basic Medicine, Gansu University of Chinese Medicine, Lanzhou 730000, China; 3 School of traditional Chinese and Western Medicine, Gansu University of Chinese Medicine, Lanzhou 730000, China; 4 School of Public Health, Gansu University of Chinese Medicine, Lanzhou 730000, China; 5 Teaching Experiment Training Center, Gansu University of Chinese Medicine, Lanzhou 730000, China

**Keywords:** type H endothelial cells, vascular–osteogenic coupling, cell signaling, senolytics, small molecule modulators, knee osteoarthritis

## Abstract

The pathogenesis of knee osteoarthritis (KOA) involves bone homeostasis imbalance induced by inflammation, metabolism, age, mechanical stress, joint injury and other factors. Recently, Type H vessels (CD31^hi^EMCN^hi^ endothelial cells) have emerged as a specialized endothelial cell subset that couples angiogenesis with osteogenesis. Type H angiogenesis in the diaphysis was found to be beneficial for maintaining bone homeostasis, while the abnormal proliferation of type H vessels in subchondral bone can lead to chondrocyte hypertrophy and osteophyte formation. However, the mechanism by which the abnormal proliferation of type H vessels induces KOA has not been elucidated in detail. In this review, we summarize the latest evidence on the role of type H endothelial cell function in the pathogenesis and progression of osteoarthritis (OA). We review the role of type H angiogenesis at different sites in the development and progression of OA and focus on the potential mechanisms that regulate type H angiogenesis in OA and the potential therapeutic value and significance of targeting type H angiogenesis in promoting bone regeneration and maintaining bone homeostasis. Finally, we discuss key obstacles and future directions for studying type H vessel regulation in KOA, offering an endothelial cell-based framework for understanding bone homeostasis and improving OA.

## 1. Introduction

Osteoarthritis (OA) is a common degenerative joint disease, but its nature is no longer viewed as simple cartilage wear and tear. Instead, it is recognized as a whole-joint disorder that involves cartilage degeneration, subchondral bone sclerosis, osteophyte formation, and pathological changes in the synovium, meniscus, and infrapatellar fat pad [1]. Data show that the global prevalence of OA increased by 48% from 1990 to 2019, accounting for 2.2% of the global disease burden and identifying it as the fourth leading cause of disability [2]. According to 2024 study data [3], the burden of OA continues to escalate globally, affecting 7.6% of the population. OA has become the seventh leading cause of disability after the age of 70 in the world, bringing a heavy economic burden to society and families. Other studies have found that OA commonly affects the knee joint, which accounts for up to 85% of the OA cases worldwide [2,4]. Regarding the pathogenesis of knee osteoarthritis (KOA), it is a complex, multifactorial whole-joint disease that involves not only alterations in bone and cartilage homeostasis, but also inflammatory and metabolic changes affecting the synovium, infrapatellar fat pad, and other joint tissues [5]. Age, sex, obesity, and mechanical stress are important risk factors [6]. Moreover, the synovium and infrapatellar fat pad act as critical sources of pro-inflammatory mediators, cytokines, and adipokines, which further contribute to cartilage degradation, subchondral bone remodeling, and pain, thereby driving the onset and progression of KOA [5]. Further studies have revealed that, as the most pro-angiogenic endothelial cell subset in bone, type H vessels (CD31^hi^EMCN^hi^) play a critical role in bone–vascular coupling [7]. During the pathological progression of KOA, abnormal hyperplasia of type H vessels in the subchondral bone is considered a key contributor to the disruption of bone homeostasis [8]. Therefore, it is urgent to explore therapeutic targets from the new perspective of type H angiogenesis regulation.

In the normal physiological state, the subchondral bone is in a state of dynamic balance, which is the basis for normal joint function. However, during the pathogenesis of OA, PDGFR-β in endothelial cells promotes aberrant type H vessel formation in the subchondral bone through the talin1/focal adhesion kinase (FAK) pathway [9]. This leads to more active subchondral bone formation, ultimately resulting in chondrocyte hypertrophy and osteophyte formation, thereby exacerbating OA progression [9]. Lu et al. used transgenic mice with chondrocyte-specific mechanistic target of rapamycin complex 1 (mTORC1) activation or inhibition, and subjected them to surgically induced OA by destabilization of the medial meniscus. They demonstrated that mTORC1 activation in chondrocytes upregulates the expression of vascular endothelial growth factor A (VEGFα), and promotes type H vessel formation in subchondral bone, thereby exacerbating articular cartilage degeneration [10]. Notably, the new type H vessels in subchondral bone deliver nutrients to the cartilage-bone junction, which reactivates mTORC1 in chondrocytes and promotes type H angiogenesis by increasing VEGFα secretion, thus forming a malignant positive feedback loop that continuously exacerbates the structural destruction and disease progression of OA [10]. Liu et al. found angiogenic chondrocyte-2 (AngC-2) in the subchondral bone of KOA mice, which can secrete fibroblast growth factor 2 (FGF2). FGF2 can promote the abnormal Type H angiogenesis of subchondral bone, leading to the occurrence of subchondral bone sclerosis and osteophyte formation in KOA [8]. In contrast, in osteoblasts, mTORc1 activation inhibits type H angiogenesis in the diaphysis by stimulating the production of VEGF and C-X-C motif chemokine ligand 10 (CXCL10) [11]. Therefore, activating mTORc1 is strictly tissue-selective in the regulation of type H angiogenesis, and the tissue-specific abnormal proliferation of type H vessels is an important factor in the occurrence and development of KOA. However, systematic analyses of the molecular mechanism by which type H angiogenesis affects the KOA process remain insufficient.

In this review, we provide a framework for understanding the role of type H angiogenesis in the development of KOA, clearly elucidating the tissue-specific regulatory characteristics of type H angiogenesis in the diaphysis and cartilage, and detailing the key mechanisms by which type H angiogenesis regulates KOA triggered by a series of axis-signaling, including hypoxia-inducible factor/vascular endothelial growth factor (HIF/VEGF), slit guidance ligand 3/roundabout axon guidance receptor (SLIT3/ROBO), delta-like canonical Notch ligand 4 (DLL4)/Notch, HIF/mammalian target of rapamycin (mTOR), HIF/SLIT, and lectin-like oxidized low-density lipoprotein receptor-1 (LOX-1)/HIF-1α/solute carrier family 7 member 11 (SLC7A11). We also summarize the potential therapeutic value and significance of the targeted regulation of type H angiogenesis in promoting bone regeneration and maintaining bone homeostasis during KOA pathogenesis. Finally, we discuss the major obstacles and future directions for regulating type H angiogenesis in KOA.

## 2. Type H Vessels and Bone

### 2.1. Physiopathology of Type H Vessels and Bone

Recent studies have reported that a special subtype of microvessels, called type H vessels, is located near the metaphyseal growth plate and diaphyseal periosteum. These microvessels highly express platelet endothelial cell adhesion molecule-1 (PLEC-1, CD31) and endomucin (EMCN) [12,13]. On the one hand, type H vessels play a role in osteogenesis by recruiting osteoprogenitor cells, promoting the differentiation of osteoblasts and osteocytes, regulating bone growth and maintaining bone dynamic balance. On the other hand, type H endothelial cells can promote angiogenesis and form a vascular network after neovascularization. By extending through the surface of bone trabeculae, periosteum, endosteum, and cortical bone, they can nourish and repair injured joints [7,14], promote the metabolism of cartilage and periarticular tissues, and finally alleviate the pathological process of cartilage degeneration and bone loss. Thus, type H vessels have the effect of coupling blood vessels and osteogenesis [9].

**Figure 1:**
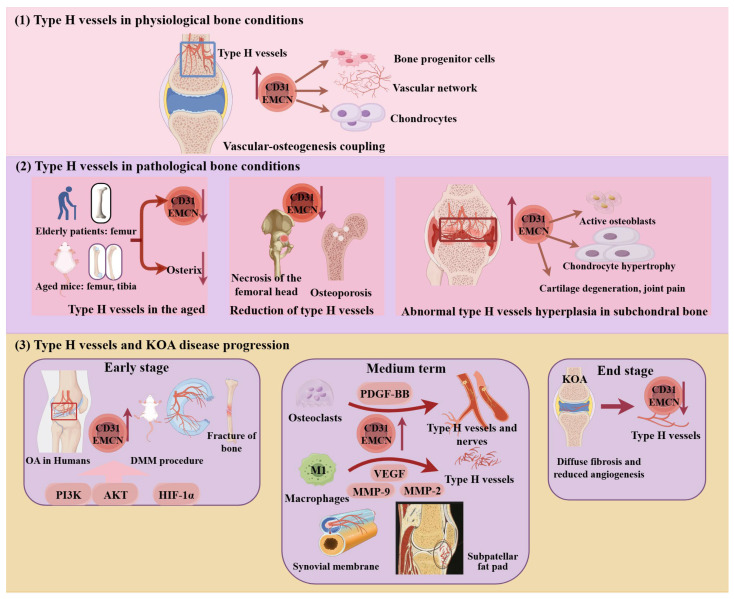
Spatiotemporal regulation of type H angiogenesis in physiological bone homeostasis and the pathological progression of knee osteoarthritis (KOA). This figure summarizes the effect of H-type angiogenesis on the progression of physiological state, chronic bone disease and KOA disease, respectively. (**1**) Physiological state. In normal joints, type H vessels (CD31^hi^EMCN^hi^) located near the epiphyseal plate and diaphyseal periosteum maintain bone homeostasis through angiogenesis–osteogenesis coupling. These vessels recruit osteoprogenitor cells, promote the differentiation of osteoblasts and osteocytes, and regulate chondrocyte function. (**2**) Age-related decline and abnormal proliferation. With aging or under pathological conditions, decreases in type H vessels are commonly observed in osteonecrosis of the femoral head and postmenopausal osteoporosis, leading to insufficient blood supply and bone loss. Conversely, the abnormal hyperplasia of type H vessels in subchondral bone is a key driver of early KOA, resulting in chondrocyte hypertrophy and osteophyte formation. (**3**) Type H vessel dysplasia and pathological progress of KOA, depicted in three stages from left to right: Early stage, HIF-1α and PI3K/AKT signaling pathways promote aberrant type H vessel proliferation in subchondral bone, initiating joint destruction in both mouse models and human OA. Intermediate stage, persistent abnormal proliferation of subchondral type H vessels is accompanied by PDGF-BB secreted by osteoclasts and VEGF/MMPs released by M1 macrophages, which disrupt the vascular barrier, induce pathological angiogenesis, and accelerate cartilage degradation and chondrocyte hypertrophy. The infrapatellar fat pad and synovium also contain rich vascular networks associated with KOA-related inflammation and fibrosis; however, whether these vessels include the type H subtype remains unclear. End stage, type H vessel density markedly declines, accompanied by subchondral bone sclerosis or cystic degeneration. The synovium exhibits diffuse fibrosis and reduced neovascularization, leading to irreversible joint degeneration, cartilage loss, and pain. Abbreviations: CD31, platelet endothelial cell adhesion molecule-1; DMM, destabilization of the medial meniscus; EMCN, endomucin; HIF-1α, hypoxia-inducible factor 1α; KOA, knee osteoarthritis; M1, classically activated macrophage; MMP, matrix metalloproteinase; PDGF-BB, platelet-derived growth factor BB; PI3K, phosphatidylinositol 3-kinase; AKT, AKT serine/threonine kinase; VEGF, vascular endothelial growth factor. This illustration was created using Figdraw (www.figdraw.com, accessed on 19 July 2026) with copyright code ASPTI54c15.

Under normal physiological conditions, type H angiogenesis is in a balanced state, and its vascular–osteogenic coupling is conducive to maintaining articular bone homeostasis. However, the abundance of type H vessels is age-dependent and decreases significantly with aging, which may lead to age-related bone loss [12,15]. Wang et al. found that type H vessels were almost absent in bone slices from elderly patients with proximal femoral fractures, and type H vessels were significantly reduced in the tibia and femur of aged mice [15]. Under pathological conditions, the abnormal number and function of type H vessels may promote the occurrence and development of diseases. Decreases in the number of type H vessels are common in glucocorticoid-induced osteonecrosis of the femoral head and postmenopausal osteoporosis. This reduction leads to local bone blood supply insufficiency and nutrient deficiency, which weakens osteogenic function and promotes bone destruction and bone loss [16,17]. However, the abnormal increase and dysfunction of type H vessels are important causes of subchondral bone destruction in OA [18]. The abnormal proliferation of type H vessels in subchondral bone makes the subchondral bone more active, eventually leading to chondrocyte hypertrophy and the formation of cartilage osteophytes, which aggravates the occurrence and development of KOA [9]. This pathological proliferation also leads to cartilage degeneration and joint pain by aggravating inflammation, nerve invasion, and cartilage destruction [18,19]. Therefore, the downregulation of CD31 and EMCN expression not only causes the imbalance of bone homeostasis in chronic bone diseases such as femoral head necrosis and osteoporosis by disrupting the vascular-bone coupling, but also plays an important role in the cartilage destruction and bone spur formation of KOA (Figure 1). Given that the reduction in the number of CD31^+^EMCN^+^ type H vessels and the decrease in Osterix (Osx) expression are important features of these pathologies, targeting the regulation of type H vessels formation holds considerable promise for the treatment of KOA (Figure 1).

### 2.2. Type H Vessels and KOA Disease Progression

Type H vessels show dynamic changes in different stages of bone disease and may aggravate disease progression. The number of type H vessels increases greatly in the early stage of KOA. In human OA studies, Li et al. demonstrated a marked increase in the number of type H vessels in the subchondral bone of OA patients, and that elevated serum leptin promotes this process via activation of the PI3K/AKT pathway [20].The number of type H vessels increases greatly in the early stage of KOA. For example, the number of type H vessels in the meniscus of OA mice induced by medial meniscus instability (DMM) was shown to be significantly higher than that of normal mice, and a large number of type H vessels were generated in the early stage (2 weeks) after DMM [10,21]. Kim et al. further found that HIF-1α was the main molecule promoting type H angiogenesis in the early stage of fracture healing (1–2 weeks) in rats, and SLIT3 was the main molecule promoting type H angiogenesis in the intermediate to late stages (4–8 weeks) [22]. Notably, in patients with end stage KOA, the synovium exhibits diffuse fibrosis and reduced neovascularization [23]. We speculate that the differential changes in type H vessels density may be closely related to the lesion site during KOA progression. To date, the pathological significance of aberrant type H vessels hyperplasia in the meniscus remains unclear. Whether it contributes to KOA progression by affecting meniscal structural integrity or the local microenvironment warrants further investigation. In a mouse model of DMM-induced OA, subchondral osteoclasts secrete platelet-derived growth factor BB (PDGF-BB) during KOA progression, which induces pathological invasion of type H vessels into articular cartilage. Aberrant sensory nerve fibers also invade along these vascular channels, leading to pain [24]. Fan et al. summarized in a review that M1 macrophages in KOA are also involved in type H angiogenesis. These macrophages secrete VEGF, matrix metalloproteinase (MMP)-9, and MMP-2, destroying the vascular barrier, enhancing the invasion of type H endothelial cells, promoting the proliferation of abnormal type H vessels, and aggravating the pathological progression of KOA [25]. It is well known that the infrapatellar fat pad contains a rich vascular network and is associated with KOA-related inflammation and fibrosis [26]. However, whether these vessels include the type H subtype (CD31^+^EMCN^+^) and whether they contribute to KOA pathology remain unclear. Similarly, although synovial angiogenesis is a recognized feature of KOA [23], direct evidence for type H vessels involvement in the synovium is still lacking. Therefore, in addition to the involvement of type H vessels in the subchondral bone and meniscus in the pathogenesis of KOA, a key question remains: do type H vessels exist in other joint tissues of KOA? Further in vitro and in vivo experiments are needed to address this question. Furthermore, the distribution characteristics of type H vessels in different joint tissues should be further elucidated, and their specific roles in the pathogenesis of KOA should be determined. Therefore, the number of type H vessels is highly correlated with the stage of KOA disease, and is regulated by different signaling molecules or macrophage polarization (Figure 1).

### 2.3. Type H Vessels in Bone Tissues Are Associated with KOA Disease

#### 2.3.1. Type H Vessels in the Diaphysis Bone

The main function of type H vessels in the metaphysis is to provide oxygen and nutrients to metaphyseal cells, thereby maintaining bone home ostasis [27]. In femoral head samples from patients with osteonecrosis of the femoral head and hip osteoarthritis, type H endothelial cell vulnerability is associated with necrosis severity, hypoxia, ferroptosis, parthanatos, increased inflammation, and altered SLIT3–ROBO4 signaling [28]. These findings indicate that the survival and function of type H endothelial cells are regulated by multiple signaling pathways, which also mediate angiogenesis–osteogenesis coupling in the diaphyseal region.

Type H vessels mediate the vascular–osteogenic coupling through multiple signaling pathways. In the ovariectomized (OVX) mouse model, platelet-derived growth factor BB (PDGF-BB) secreted by preosteoclasts is a key molecule that induces type H vessel formation in the femur [29]. Nuciferine could inhibit mitogen-activated protein kinase (MAPK) and NF-κB signaling pathways, retain tartrate-resistant acid phosphatase^+^ (TRAP^+^) preosteoclasts, promote PDGF-BB secretion, and increase the number of type H vessels [30]. Other studies found that type H vessels can mediate their coupling with osteogenesis through HIF-1α, VEGF, and Notch signaling pathways [7,16]. Notably, Sirtuin 1 (SIRT1) promotes type H vessel formation and osteogenesis by activating the Phosphatidylinositol 3-Kinase (PI3K)/AKT serine/threonine kinase (AKT)/Forkhead box protein O1 (FOXO1) signaling pathway, a mechanism that has been validated in both femoral and mandibular defect mouse models [31], suggesting that the core regulatory pathways of type H vessels may be shared between long bones and craniofacial bones.

**Figure 2:**
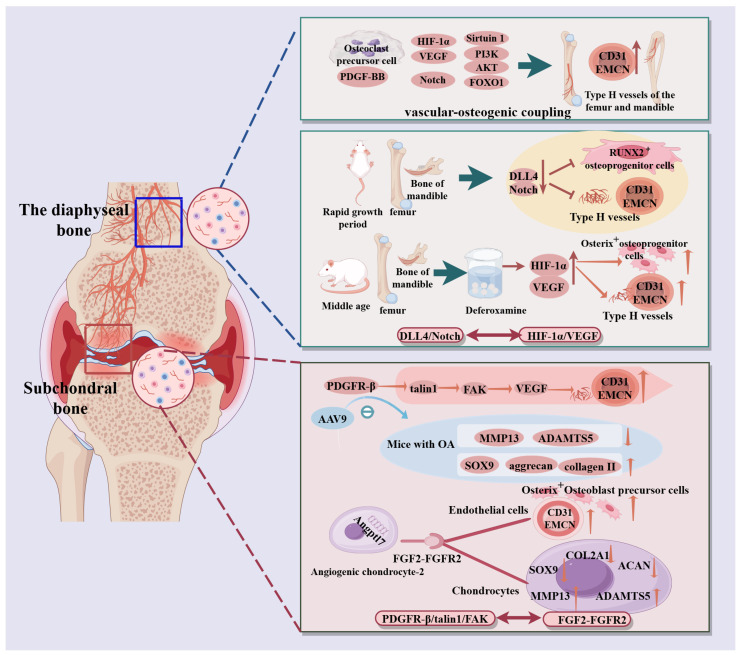
Key regulatory mechanisms of type H vessels in the diaphysis and subchondral bone. The figure is divided into two tiers, contrasting the regulatory mechanisms of type H vessels (CD31^hi^EMCN^hi^) in the diaphysis (upper) and subchondral bone (lower). (Upper tier) Diaphyseal bone. In the femur and tibia, key molecules involved in angiogenesis–osteogenesis coupling include PDGF-BB secreted by preosteoclasts, HIF-1α/VEGF, Notch, and the Sirtuin 1/PI3K/AKT/FOXO1 pathway. In the femur and mandible, type H vessels are regulated by stage-dependent signaling pathways, exhibiting a functional transition from the Notch pathway in adolescence to the HIF-1α pathway in adulthood, thereby maintaining angiogenesis–osteogenesis coupling and bone homeostasis. (Lower tier) Subchondral bone. In KOA subchondral bone, pathological signaling pathways drive aberrant type H vessel proliferation and cartilage degeneration. The PDGFR-β/talin1/focal adhesion kinase (FAK) signaling axis is activated in endothelial cells, promoting type H angiogenesis. Local injection of adeno-associated virus serotype 9 (AAV9) into subchondral bone to specifically inhibit endothelial PDGFR-β reduces the secretion of the degradative enzymes MMP13 and ADAMTS5, while upregulating SOX9, aggrecan, and collagen II. Furthermore, a newly identified angiogenic chondrocyte subpopulation, angiogenic chondrocyte-2, secretes FGF2, which activates FGFR2 on endothelial cells, further promoting pathological type H vessel formation. These pathological alterations lead to chondrocyte hypertrophy, cartilage degeneration, and joint pain. Abbreviations: AAV9, adeno-associated virus serotype 9; ACAN, aggrecan; ADAMTS5, a disintegrin and metalloproteinase with thrombospondin motifs 5; AKT, AKT serine/threonine kinase; CD31, platelet endothelial cell adhesion molecule-1; DLL4, delta like canonical Notch ligand 4; EMCN, endomucin; FAK, focal adhesion kinase; FGF2, fibroblast growth factor 2; FGFR2, fibroblast growth factor receptor 2; FOXO1, forkhead box protein O1; HIF-1α, hypoxia-inducible factor 1α; MMP13, matrix metalloproteinase 13; Notch, Notch receptor; PDGF-BB, platelet-derived growth factor BB; PDGFR-β, platelet-derived growth factor receptor beta; PI3K, phosphatidylinositol 3-kinase; RUNX2, runt-related transcription factor 2; SOX9, SRY-box transcription factor 9; VEGF, vascular endothelial growth factor. This illustration was created using Figdraw (www.figdraw.com, accessed on 19 July 2026) with copyright code WRPTSc444f.

Based on this understanding, the condyle, as a bony structure of the mandible, may serve as a reference for understanding the regulatory mechanisms of type H vessels in the diaphysis. Using the femur and mandible as a model, Hu et al. found that deferoxamine mesylate promoted type H angiogenesis by activating the HIF-1α signaling pathway, which then recruited Osx^+^ osteoprogenitor cells to the condyle and significantly enhanced mandibular advancement (MA)-induced condylar osteogenesis, especially in middle-aged mice in which bone growth had been arrested [32]. Hu et al. further studied 6-week-old male mice (rapid growth phase, MA-sensitive) and found that inhibiting the DLL4/Notch signaling axis reduced type H angiogenesis, which, in turn, reduced the number of early runt-related transcription factor 2^+^ (RUNX2^+^) osteoprogenitor cells and impaired MA-induced condylar osteogenesis, as well as resulted in shortened femurs and reduced bone mass [33]. Therefore, the signaling axes regulating type H vessel formation exhibit marked heterogeneity across different skeletal sites and developmental stages. In the diaphysis of long bones, HIF-1α, VEGF, Notch, PDGF-BB, and SIRT1 pathways collectively participate in vascular–osteogenic coupling (Figure 2). In the craniofacial condyle, these pathways display distinct stage-dependent heterogeneity, manifested as a functional transition from the Notch pathway in adolescence to the HIF-1α pathway in adulthood, which may provide new insights for the treatment of OA (Figure 2).

#### 2.3.2. Type H Vessels in Subchondral Bone

Type H vessel dysplasia is considered to be an important link between cartilage degeneration and bone remodeling [18,25,34,35]. Under normal circumstances, type H vessels in the metaphysis provide oxygen and nutrition, which are beneficial to bone repair. Type H vessels not only have a unique structure but also have the dual functions of promoting angiogenesis and promoting bone formation. Previous studies found that type H vessels were significantly reduced in the metaphyseal region of aged mice, while type H vessels were significantly increased in subchondral bone, suggesting that the number and role of type H vessels are different at different locations [10]. The abnormal proliferation of type H vessels in subchondral bone leads to more active subchondral osteogenesis, eventually leading to chondrocyte hypertrophy and osteophyte formation, and aggravating progression of the disease [9,18,36]. Therefore, inhibiting the abnormal proliferation of type H vessels in subchondral bone and increasing the number of type H vessels in the metaphyseal area are crucial for preventing and treating OA.

Under normal physiological conditions, mature cartilage is devoid of blood vessels and nerves. However, in OA patients, type H vessels infiltrate the tidal line and invade the articular cartilage through vertical microchannels, breaking the homeostasis of articular cartilage [24]. Type H vessels invading the subchondral bone can promote inflammatory responses and further aggravate OA pain [37,38]. A number of studies have shown significant increases in type H vessels in the subchondral bone of OA mice [9,37,39]. Cui et al. studied OA mice established by anterior cruciate ligament transection (ACLT). Endothelium-specific enhanced platelet-derived growth factor receptor beta (PDGFR-β) was reported to promote type H angiogenesis in subchondral bone by activating the talin 1/FAK signaling pathway in these mice, leading to subchondral bone and cartilage lesions, thereby driving OA malignant progression [9]. Notably, a subchondral bone injection of adeno-associated virus 9 (AAV9), which specifically inhibits endothelial PDGF-β, effectively alleviated OA [9]. Therefore, PDGFR-β has become a new target for OA treatment. Another study found that AngC-2, highly expressing angiopoietin-like protein 7 (Angptl7) is present in KOA mice, driving abnormal type H angiogenesis in subchondral bone by secreting FGF2 to activate fibroblast growth factor receptor 2 (FGFR2) in endothelial cells and promote cartilage matrix degradation in KOA [8]. Inhibiting Angptl7 or blocking FGF2-FGFR2 signaling can alleviate the progression of KOA, providing new ideas and strategies for targeting cartilage-vascular crosstalk [8]. However, the mechanism of type H vessel abnormal proliferation in subchondral bone has only been studied in animal experiments and cell experiments, and no in-depth clinical trial study has been conducted. In summary, in the OA pathological microenvironment, type H vessel dysplasia is regulated by multiple signal axes such as FGF2-FGFR2 and PDGFR-β, which participate in the balance between cartilage metabolism and angiogenesis by affecting MMP13, a disintegrin and metalloproteinase with thrombospondin motifs 5 (ADAMTS5) and other factors (Figure 2). This provides a rationale for targeting this signaling network to restore vascular-cartilage homeostasis.

#### 2.3.3. Mechanistic Basis for the Functional Differences in Type H Vessels in the Diaphysis and Subchondral Bone

Type H vessels exhibit opposing functional phenotypes in the diaphysis and subchondral bone, and this tissue-specific duality likely arises from differences in the microenvironment, cellular composition, and mechanical loading between these two regions.

From the microenvironmental perspective, the diaphyseal region is characterized by physiological hypoxia and mechanical stimulation, which maintain moderate vascular–osteogenesis coupling [7]. In contrast, subchondral bone is characterized by exacerbated hypoxia and elevated inflammatory factors under OA pathological conditions, driving aberrant type H vessel proliferation and bone destruction [8,10]. From the cellular composition perspective, perivascular cells in the diaphysis, such as pericytes and osteoprogenitor cells, maintain vascular stability and moderate osteogenic coupling through PDGF-BB and SIRT1 signaling [29,31]. Conversely, the subchondral bone is characterized by excessive osteoclast activation [24], M1 macrophage infiltration [25], and the emergence of Angptl7^+^ angiogenic chondrocytes [8], which collectively constitute a pro-angiogenic and pro-inflammatory microenvironment. From the mechanical loading perspective, the diaphysis is subjected to physiological axial stress, and physiological mechanical stimulation promotes type H angiogenesis and bone repair [32]. In contrast, the subchondral bone in OA experiences abnormal shear stress and local stress concentration, which drive aberrant type H vessel proliferation through the PDGFR-β/talin1/FAK axis [9]. In summary, the functional differences in type H vessels in the diaphysis and subchondral bone can be attributed to three levels of mechanistic divergence: the microenvironment (hypoxic state and mechanical stimulation), cellular composition (perivascular cell types and immune status), and mechanical loading (physiological versus pathological). This tissue-specific regulatory framework provides a biological basis for developing spatially precise therapeutic strategies.

Although the aforementioned studies have revealed the dynamic changes in type H vessels at different sites and stages of KOA, the current evidence still has limitations and controversies. Regarding model selection, DMM and ACLT, as the most commonly used surgical KOA models, differ in their induction mechanisms: DMM mimics chronic joint instability, whereas ACLT induces acute joint instability. This difference may lead to variations in the time window, severity, and molecular mechanisms of type H vessel changes. Furthermore, differences in baseline type H vessels levels between mice and rats, as well as among animals of different ages, increase the complexity of cross-study comparisons. Currently, most studies employ only a single model and lack multi-model cross-validation. Regarding spatiotemporal heterogeneity, type H vessels increase in early KOA but may decrease in the end stage. However, there is no unified standard for defining disease stages across studies, and most studies only observe a single time point, failing to fully capture the dynamic evolution of type H vessels. Moreover, type H vessels exhibit opposing regulatory trends in the diaphysis and subchondral bone, yet no study has simultaneously analyzed both sites, leading to insufficient evidence. Notably, current research on type H vessels in KOA has predominantly focused on subchondral bone, where their aberrant proliferation has been extensively documented. In contrast, the changes in type H vessels within the diaphyseal region during KOA remain largely uninvestigated. Existing evidence only indicates that type H vessels decline with aging [15], yet whether age-related vascular regression and OA pathology exert superimposed or synergistic effects remains unclear. This gap in evidence further limits a comprehensive understanding of the functional differences in type H vessels across distinct skeletal sites. Regarding methodology, the quantitative analysis of type H vessels primarily relies on CD31/EMCN immunofluorescence semi-quantitative assessment, which cannot fully reflect the three-dimensional structure of the vasculature. Most human OA specimens are derived from end-stage samples obtained after joint replacement surgery, making it difficult to reflect pathological changes in early and middle stages. Additionally, some interventional studies have only been validated in non-OA models such as osteoporosis and bone defects, and their applicability in KOA remains to be confirmed.

In summary, future research should focus on the following directions: (1) adopting multi-timepoint and multi-model experimental designs to systematically map the dynamic changes in type H vessels at different stages of KOA; (2) conducting cross-validation across multiple OA models, including DMM, ACLT, and monosodium iodoacetate, as well as across different species (mice, rats, humans) to enhance the robustness of conclusions; (3) integrating high-throughput technologies such as single-cell transcriptome sequencing and spatial transcriptomics to deeply dissect the molecular heterogeneity of type H endothelial cells and their communication networks with surrounding cells (e.g., osteoclasts, macrophages, chondrocytes); (4) separately sampling and independently analyzing type H vessels in the diaphysis and subchondral bone to clarify their tissue specificity; (5) Investigating the changes in type H vessels within the diaphyseal region during KOA and elucidating the interplay between age-related vascular regression and OA pathology; and (6) conducting clinical studies with early-stage OA samples to provide clinical validation for findings from animal experiments. Through these efforts, the field may advance from “phenomenon description” to “mechanistic elucidation” and provide an evidence base for the spatiotemporally precise regulation of type H vessels.

## 3. Regulatory Mechanism of Type H Angiogenesis in Knee OA

**Figure 3:**
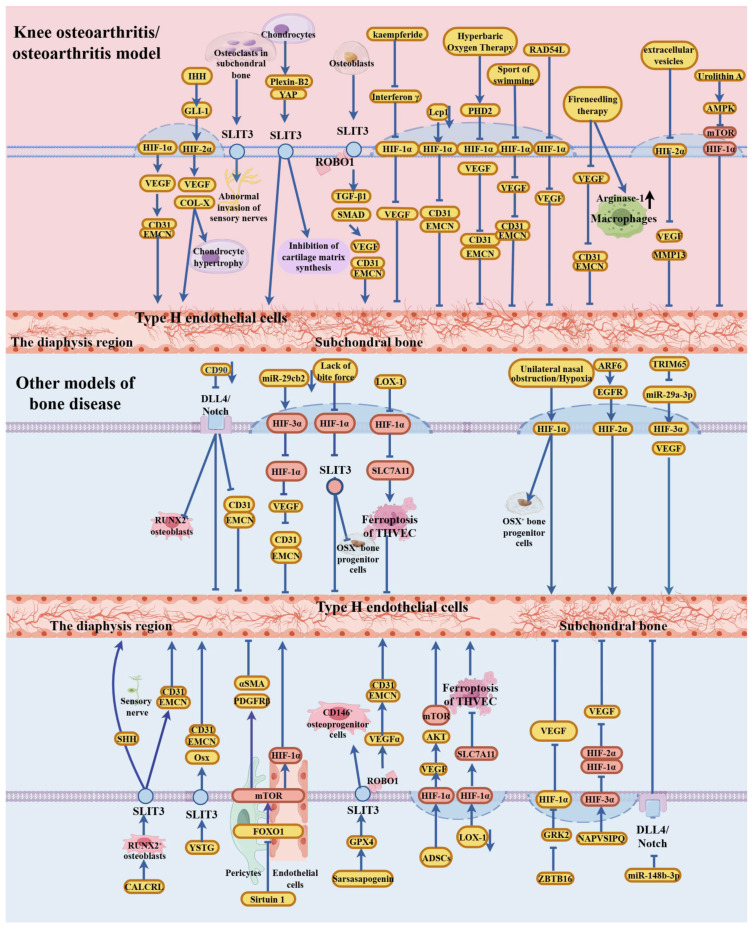
Bidirectional regulation of type H angiogenesis by multiple signaling axes in the knee osteoarthritis microenvironment. The pathogenesis of knee osteoarthritis (KOA) is characterized by distinct spatiotemporal alterations in type H vessels within the diaphysis and subchondral bone regions. (Top panel, red) In KOA/OA models, aberrant activation of signaling pathways—including HIF/VEGF, SLIT3, and HIF/mTOR—drives pathological proliferation of type H vessels (CD31^hi^EMCN^hi^) in subchondral bone, leading to abnormal angiogenesis–osteogenesis coupling, sensory nerve invasion, and cartilage matrix degradation. Therapeutic interventions (e.g., hyperbaric oxygen therapy, fire needle therapy) target these axes to restore homeostasis. (Bottom panel, blue) In other bone disease models or reparative states, signaling axes such as HIF/VEGF, SLIT, DLL4/Notch, HIF/mTOR, HIF/SLIT, and LOX-1/HIF-1α/SLC7A11 regulate osteoprogenitor cell differentiation and pericyte function, promoting physiological type H vessel formation and bone repair. Red nodes indicate key crosstalk points or therapeutic targets within the signaling network. This bidirectional regulatory mechanism highlights the complexity of the bone microenvironment and offers precise therapeutic strategies for KOA. Abbreviations: ADSCs, adipose-derived stem cells; AMPK, AMP-activated protein kinase; ARF6, ADP-ribosylation factor 6; CALCRL, calcitonin receptor-like receptor; CD31, platelet endothelial cell adhesion molecule-1; CD90, cluster of differentiation 90; CD146, cluster of differentiation 146; COL-X, collagen type X; DLL4, delta-like ligand 4; EGFR, epidermal growth factor receptor; EMCN, endomucin; FOXO1, forkhead box O1; GLI-1, glioma-associated oncogene homolog 1; GPX4, glutathione peroxidase 4; GRK2, G protein-coupled receptor kinase 2; HIF-1α, hypoxia-inducible factor 1-alpha; HIF-2α, hypoxia-inducible factor 2-alpha; HIF-3α, hypoxia-inducible factor 3-alpha; IHH, Indian hedgehog; Lcp1, lymphocyte cytosolic protein 1; LOX-1, lectin-like oxidized low-density lipoprotein receptor-1; miR-29a-3p, microRNA-29a-3p; miR-29cb2, microRNA-29cb2; miR-148b-3p, microRNA-148b-3p; mTOR, mechanistic target of rapamycin; NAPVSIPQ, Asn-Ala-Pro-Val-Ser-Ile-Pro-Gln; Notch, Notch receptor; OA, osteoarthritis; Osx, Osterix; PDGFR-β, platelet-derived growth factor receptor beta; PHD2, prolyl hydroxylase domain-containing protein 2; Plexin-B2, plexin B2; RAD54L, RAD54-like; ROBO1, roundabout guidance receptor 1; RUNX2, runt-related transcription factor 2; SHH, sonic hedgehog; SLC7A11, solute carrier family 7 member 11; SLIT3, slit guidance ligand 3; TGF-β1, transforming growth factor beta 1; THVEC, type H vascular endothelial cell; TRIM65, tripartite motif-containing protein 65; VEGF, vascular endothelial growth factor; VEGFα, vascular endothelial growth factor A; YAP, Yes-associated protein; YSTG, Yi Shen Tiao Gan decoction; ZBTB16, zinc finger and BTB domain-containing protein 16; αSMA, alpha-smooth muscle actin. This illustration was created using Figdraw (www.figdraw.com, accessed on 19 July 2026) with copyright code PIAOReeee4.

Type H vessels are coupled to osteoblasts, a phenomenon that suggests the complexity of the molecular regulatory network between endothelial cells and osteoblasts. So far, several important factors regulating type H vessels and osteogenesis have been identified [7,18,35]. Osteoclasts, osteoblasts, chondrocytes, and endothelial progenitor cells all secrete specific factors that induce endothelial cell proliferation and angiogenesis through HIF-1/VEGF, SLIT/ROBO, and DLL4/Notch signaling axes, as well as through HIF/mTOR, HIF/SLIT, and LOX-1/HIF-1α/SLC7A11 crosstalk signaling axes [7,35] (Figure 3). There may also be other regulators that have not yet been elucidated (Figure 3). Table 1 summarizes the specific mechanisms of action of the above signaling axes in the coupling of type H vessels to osteogenesis.

### 3.1. HIF/VEGF Signaling Axis

Hypoxia-inducible factors, belonging to the basic helix–loop–helix/Per-Arnt-Sim (bHLH-PAS) family, are key regulators of the hypoxic response. They consist of an oxygen-sensitive alpha subunit and a stable HIF-1β subunit. In OA, HIF-α isoforms exhibit distinct functions: HIF-1α is upregulated in OA chondrocytes and maintains cartilage matrix stability via anabolic pathways, whereas HIF-2α induces chondrocyte apoptosis and inflammation, playing a catabolic role [56,57]. VEGF, a classical HIF-1 target, is abnormally elevated in KOA patients, with serum levels positively correlating with disease severity [58,59]. Using male mice with sphingosine-1-phosphate receptor 3 knockout, pharmacological inhibitors, and in vitro cell culture models, it was demonstrated that osteoblasts secrete VEGF via the sphingosine-1-phosphate/sphingosine-1-phosphate receptor 3 pathway, promoting their own mineralization through an autocrine mechanism and enhancing bone formation through type H angiogenesis via a paracrine mechanism [60]. Wei et al. demonstrated that fire needle therapy reduces subchondral bone destruction by reducing type H vessels, VEGF, and pro-inflammatory macrophages in a monosodium iodoacetate-induced KOA rat model [61]. Thus, VEGF-regulated type H angiogenesis has dual roles: promoting osteogenic repair in the diaphysis while contributing to subchondral bone pathology. Future therapies may target the HIF-1/VEGF axis to promote repair in the diaphysis and inhibit angiogenesis in subchondral bone.

#### 3.1.1. HIF-1α/VEGF

HIF-1α is central to OA progression. Its abnormal activation promotes NOD-like receptor family pyrin domain-containing protein 3 inflammasome-mediated pyroptosis and Interleukin-1 beta (IL-1β)/IL-18 release, exacerbating synovial inflammation, cartilage degeneration, and fibrosis [62,63]. Conversely, zinc finger and BTB domain-containing 16 overexpression inhibits the G protein-coupled receptor kinase 2 (GRK2)/HIF-1α pathway, reducing pathological angiogenesis, inflammatory factors, and matrix metalloproteinases in collagen-induced arthritis mice [64]. Similarly, the flavonoid kaempferide alleviates OA by inhibiting the interferon-γ/HIF-1α axis, as shown in vitro and in papain-induced rat models [65]. Thus, targeting the GRK2/HIF-1α and interferon-γ/HIF-1α axes may delay OA progression by mitigating inflammation and cartilage degradation.

In recent years, studies have focused on regulating subchondral type H vessels to treat OA [9,10]. In human OA subchondral bone tissue, HIF-1α levels are significantly elevated in sclerotic subchondral bone [66], and this aberrant hypoxic microenvironment drives type H angiogenesis. Most research centers on the HIF-1α/VEGF axis. In a papain-induced KOA mouse model, the HIF-1α/VEGF pathway was activated in the subchondral bone of the tibial plateau, accompanied by increased type H vessels and cartilage degeneration. Conversely, swimming exercise downregulates the HIF-1α/VEGF, reduces type H vessels (CD31^hi^EMCN^hi^) formation in subchondral bone of knee joint, and improves cartilage and motor function [40]. Hyperbaric oxygen upregulates prolyl hydroxylase domain-containing protein 2 (PHD2) to degrade HIF-1α, thereby reducing type H endothelial cells (CD31^+^EMCN^+^) in subchondral bone and delaying osteoarthritis progression in a DMM-induced mouse model [41]. Furthermore, RAD54-like (RAD54L) overexpression suppressed the HIF-1α/VEGF signaling pathway, attenuated IL-1β-induced apoptosis in human chondrocytes in vitro, and alleviated cartilage degeneration while reducing Osteoarthritis Research Society International (OARSI) scores in a papain-induced OA rat model in vivo [42].

Notably, the regulatory mechanisms of the HIF-1α/type H vessels axis may be shared between long bones and craniofacial bones. In a unilateral nasal obstruction-induced systemic hypoxia mouse model, HIF-1α expression was upregulated in the subchondral bone of the mandibular condyle, accompanied by increased type H vessels and Osx^+^ osteoprogenitor cells [67,68] (Table S1). Although these findings originate from craniofacial models, the hypoxia/HIF-1α/type H vessel regulatory mechanisms they reveal share similarities with the pathological changes in KOA subchondral bone. Whether systemic or local hypoxia promotes aberrant type H vessel proliferation in knee subchondral bone via the HIF-1α/VEGF axis warrants further investigation. Therefore, targeting pathological type H angiogenesis via the HIF-1α/VEGF axis, improving blood oxygen circulation, and leveraging molecules such as RAD54L or exosomes represent promising strategies for OA treatment.

In addition, the specific hypoxic microenvironment in articular cartilage is crucial for HIF-1α-mediated metabolic homeostasis [43,57,69]. However, abnormal type H vessel proliferation disrupts this balance by increasing local oxygen concentrations, leading to HIF-1α degradation in chondrocytes and accelerating OA progression, as demonstrated in the ACLT mouse model and lymphocyte cytosolic protein 1 knockout studies [43]. Additionally, in a lumbar facet joint osteoarthritis mouse model, hypoxia-treated adipose-derived mesenchymal stem cell-derived exosomes inhibited type H endothelial cell (CD31^+^EMCN^+^) formation in subchondral bone and alleviated joint pain, suggesting that the HIF-1α/type H vessel axis may share common regulatory mechanisms across different joint sites [70]. The above evidence indicates that HIF-1α does not simply play a protective or pathogenic role in KOA, but rather exhibits spatial, temporal, and cell-type dependency. From the spatial dimension, HIF-1α promotes angiogenesis–osteogenesis coupling in the diaphyseal region, whereas its excessive accumulation in subchondral bone drives pathological vascular invasion. Interventions such as hyperbaric oxygen therapy and swimming exercise can reduce pathological type H vessel proliferation by inhibiting the subchondral HIF-1α pathway. From the temporal dimension, HIF-1α promotes aberrant type H vessel proliferation in early KOA, while its sustained overactivation in the late stage accelerates cartilage degeneration. From the cell-type dimension, the functional differences in HIF-1α across different cell types may further contribute to the complexity of its regulation. Therefore, precisely targeting HIF-1α requires comprehensive consideration of the interactive effects of spatial, temporal, and cell-type dimensions.

#### 3.1.2. HIF-2α/VEGF

HIF-2α and HIF-1α have similar structures and are both regulated by oxygen concentrations in OA. However, compared with HIF-1α anabolism, which protects articular cartilage, HIF-2α has a catabolic effect on articular cartilage, which promotes the destruction of cartilage and the progression of OA [56]. Studies have found that the abnormally high expression of HIF-2α in OA can activate a variety of pathogenic genes, such as cartilage hypertrophy markers collagen type X alpha 1 chain (COL10A1), MMP13, and VEGF, which synergistically promote cartilage matrix degradation, calcification, and pathological vascular invasion, and eventually destroy articular cartilage. The expression level of HIF-2α in the early and middle stages of OA is positively correlated with the progression of the disease. Inhibiting HIF-2α can effectively prevent the occurrence of OA [71,72]. Song et al. showed that the dietary supplement *Latilactobacillus sakei* LB-P12 also inhibited the nuclear factor kappa B (NF-kB)/HIF-2α signaling pathway, reduced inflammatory cytokines (IL-6), and downregulated the expression of *endothelial PAS domain protein 1* (the gene encoding HIF-2α) and *MMP13* in chondrocytes. These effects alleviated cartilage degradation and joint destruction in OA rats [44]. The findings provide a new idea for intervention based on the gut– joint axis. In summary, HIF-2α promotes cartilage matrix degradation, calcification, and pathological vascular invasion, destroying articular cartilage. Thus, inhibiting HIF-2α could be a focus of targeted therapy in the future.

Studies have further confirmed that phosphorylated HIF-2α can upregulate the expression of VEGF, and the high expression of VEGF binds to VEGF receptor 2 on adjacent endothelial cells to promote angiogenesis [73]. Zhang et al. demonstrated that the Indian hedgehog (IHH)-glioma-associated oncogene homolog-1 (GLI-1)-HIF-2α positive feedback axis forms in hypertrophic chondrocytes in a DMM-induced early OA mouse model. This axis drives subchondral bone vascular invasion and cartilage matrix breakdown in early OA by stabilizing HIF-2α expression and upregulating VEGF and type X collagen (COL X) expression [45]. In contrast, Mao et al. demonstrated in a unilateral anterior crossbite-induced temporomandibular joint osteoarthritis (TMJ-OA) rat model that human dental follicle cell-derived small extracellular vesicles effectively protected temporomandibular joint cartilage from OA damage by inhibiting HIF-2α and its downstream MMP13 and VEGF, which may be a new approach for effective treatment of TMJ-OA [46]. Some studies have also confirmed that IHH is an important upstream regulator of HIF-2α in OA, and the specific knockout of IHH can effectively block the abnormal angiogenesis of subchondral bone caused by the HIF-2α/VEGF pathway, thereby delaying OA progression [45]. ADP-ribosylation factor 6 (ARF6) is another key upstream regulator of HIF-2α activation. ARF6 activates HIF-2α by binding to pulmonary arterial hypertension-related model epidermal growth factor receptor (EGFR) and drives vascular remodeling [74]. Chlortetracycline, a novel ARF6 inhibitor, can downregulate HIF-2α by inhibiting the binding of ARF6 to EGFR, and could be a potential therapeutic agent [74]. However, whether the mechanism of ARF6-mediated HIF-2α activation also plays a role in OA is still unclear. In summary, the targeted regulation of the upstream factors of HIF-2α (IHH, ARF6) and the HIF-2α-related signaling axis could be a new strategy for treating OA.

#### 3.1.3. HIF-3α/VEGF

Both HIF-1α and HIF-2α are expressed in normal human OA chondrocytes and are structurally similar. In contrast, the structure of HIF-3α lacks the complete C-terminal transactivation domain while retaining the basic helix–loop–helix/Per-Arnt-Sim domains [75]. Notably, alternative splicing of the HIF-3α gene can generate a variety of negative repressor variants, such as inhibitory PAS proteins and neonatal/embryonic PAS protein, which inhibit the normal activity of the HIF pathway by competitively binding to HIF-1α/2α [76,77,78]. Some studies have suggested that HIF-3α may inhibit HIF-1α and regulate the expression of HIF-2α chondrocyte hypertrophy differentiation-related genes *COL10A* and *MMP13* [56,57]. HIF-3α may also play a negative regulatory role as a downstream target molecule of HIF-1α and HIF-2α [56,57].

Early studies found that HIF-3α is highly expressed in healthy human articular chondrocytes and negatively correlates with hypertrophic markers COL10A1 and MMP13. In contrast, HIF-3α expression is markedly reduced in OA chondrocytes, accompanied by increased COL10A1 and MMP13 expression, suggesting that low HIF-3α levels may reflect the hypertrophic state of chondrocytes [79]. However, other evidence indicates that microRNA-210 (miR-210) expression is significantly lower in cultured human OA chondrocytes compared with normal chondrocytes [47]. Overexpression of miR-210 directly targets and inhibits HIF-3α, promoting OA chondrocyte proliferation, increasing COL1A1 expression, reducing COL10A1 and MMP13 levels, and facilitating extracellular matrix deposition, thereby improving OA [47]. This finding appears contradictory to the aforementioned conclusion, which may be attributed to two factors: first, the previous study measured total HIF-3α mRNA levels, whereas the latter detected HIF-3α protein expression, representing different detection levels. Second, the HIF-3α gene can generate multiple splice variants, which may exert distinct regulatory functions. Therefore, the specific role of HIF-3α in OA needs to be evaluated based on the detection level and specific splice variants. Yang et al. also found that miR-210-3p inhibited HIF-3α expression, which significantly increased the expression of SRY-box transcription factor 9 (SOX9) and collagen type II in rat bone marrow mesenchymal stem cells, enhanced their chondrogenic ability, and downregulated peroxisome proliferator-activated receptor gamma and lipoprotein lipase, inhibiting their differentiation into adipocytes [48]. This suggests targeting this mechanism as a promising therapeutic strategy and provides an experimental basis for enhancing the cartilage-regenerative ability of OA patients. Notably, the regulatory mechanisms of the HIF-3α/type H vessel axis may be shared across different joint sites. In a lumbar facet joint osteoarthritis model, Xie et al. used exosomes to deliver miR-210-3p to inhibit HIF-3α in chondrocytes, improving articular cartilage structure and increasing matrix deposition [49], further confirming the protective role of exosome-mediated miR-210-3p/HIF-3α axis in joint diseases.

HIF-3α plays a complex role in estrogen deficiency, hypoxia, and inflammatory microenvironment. In non-OA disease models, the pro-angiogenic or anti-angiogenic functions of HIF-3α have been preliminarily revealed. In an OVX-induced osteoporosis mouse model, Ou et al. found that downregulating bone-specific miR-29cb2 in the OVX mouse model increased the expression of HIF-3α, which competitively binds to HIF-1α and inhibits the activation of HIF-1α, leading to decreased VEGF and other pro-angiogenic factors, and impaired the generation of CD31hiEMCNhi type H vessels. The recruitment and differentiation of osteoblasts were reduced, and the ability of bone formation was weakened [80]. Changes in the miR-29cb2/HIF-3α/HIF-1β axis are also present in osteoporosis patients [80]. The expression levels of HIF-3α and VEGF are significantly upregulated in the gingival crevicular fluid of periodontitis patients, suggesting that HIF-3α may be involved in local vascular reactions. However, whether HIF-3α directly regulates blood vessels has not been verified experimentally [81]. Recent studies have found that tripartite motif-containing protein (TRIM65) in retinal endothelial cells promotes pathological neovascularization by inhibiting miR-29a-3p and upregulating HIF-3α and VEGFα under high glucose stimulation [82]. NAPVSIPQ, a neuroprotective peptide, plays a role in anti-pathological angiogenesis by upregulating HIF-3α and inhibiting the HIF-1α/HIF-2α-VEGF axis [83]. These results suggest that HIF-3α can act as either a pro-angiogenic factor or a negative feedback inhibitor of angiogenesis, and its function depends on the cell type, splicing subtype, and the pathological stage. However, in KOA, the regulatory role of HIF-3α on the VEGF pathway and its relationship with aberrant type H vessels (CD31^+^EMCN^+^) proliferation remain unclear. HIF-3α is speculated to play a dual role in the regulation of KOA, and may participate in pathological angiogenesis through miR-29a-3p/TRIM65 and other mechanisms, and exert an anti-angiogenic effect by upregulating HIF-3α and inhibiting the HIF-1α/HIF-2α-VEGF signaling axis. This speculation awaits experimental validation in KOA models.

### 3.2. SLIT3/ROBO Signaling Axis

In recent years, pathological neural remodeling molecular pathways have been shown to play an important role in OA pain, which may be dependent on the synergistic growth of nerves and blood vessels, and driven by the abnormal reactivation of various guidance molecules. These pathways include VEGF, ephrins, semaphorins and SLIT3 proteins. The expression of these molecules is upregulated in OA, suggesting that they may be potential targets for novel analgesic therapies [84]. Among them, the regulatory role of SLIT3 in OA may vary depending on the tissue environment and disease stage.

In human OA studies, Li et al. further confirmed that the high expression of SLIT3 in subchondral bones of OA patients was positively correlated with VEGF expression [50]. In vitro experiments confirmed that osteoblast-derived SLIT3 combined with ROBO1 to activate the transforming growth factor beta1 (TGF-β1)/Sma- and Mad-related proteins (SMAD) pathway, the migration and angiogenesis of endothelial progenitor cells (EPCs) were enhanced by up-regulating the expression of VEGF, CD31, and EMCN [50]. In an ACLT-induced OA mouse model, intra-articular injection of sh-SLIT3 lentivirus (knocking down SLIT3) reduced OARSI scores, and decreased the number of CD31^+^EMCN^+^ endothelial cells [50]. In a TMJ-OA rat model, Zhu et al. found that activated osteoclasts in subchondral bone secrete large amounts of SLIT3, which promotes the abnormal invasion of sensory nerves into subchondral bone and aggravating pain and joint degeneration [51]. Chen et al. found that the Plexin-B2/Yes-associated protein (YAP) pathway was activated and SLIT3 expression was upregulated in TMJ-OA chondrocytes. The upregulated SLIT3 expression further promoted subchondral type H angiogenesis while inhibiting cartilage matrix synthesis, thereby driving the dual pathological process of TMJ-OA [52]. The above evidence indicates that SLIT3 exacerbates joint degeneration and pain in OA by promoting aberrant type H angiogenesis and sensory nerve invasion.

In non-OA disease models, SLIT3 exerts pro-angiogenic and osteogenic reparative effects. Osteoblast-derived SLIT3 promotes the formation of CD31^hi^EMCN^hi^ endothelial cells [85]. SLIT3 deletion leads to reduced type H vessels and decreased bone mass, whereas recombinant SLIT3 enhances fracture healing and counteracts bone loss in OVX mice [85]. Furthermore, osteoclast-derived SLIT3 stimulates osteoblast migration and proliferation by activating β-catenin, while inhibiting osteoclast differentiation in an autocrine manner [86]. In an OVX-induced osteoporosis mouse model, injection of recombinant SLIT3 significantly alleviated bone loss [86]. In postmenopausal women, higher SLIT3 levels were associated with increased bone mass [86]. Zhao et al. demonstrated in a mouse tendon–bone healing model that the calcitonin receptor-like (CALCRL) upregulates SLIT3 in RUNX2+ osteoblasts, enhancing H-type vasculature and Sonic Hedgehog (SHH) expression to promote sensory innervation and bone repair [87]. In osteoporosis models, Yi Shen Tiao Gan decoction (YSTG) increases type H vessels and osteoprogenitor cells by upregulating SLIT3 expression, thereby improving aromatase inhibitor-associated bone loss [88]. The natural compound sarsasapogenin activates glutathione peroxidase 4 (GPX4), upregulates SLIT3 in bone marrow mesenchymal stem cells (BMSCs), and subsequently activates ROBO1 in HUVECs, promoting vascular–osteogenesis coupling and alleviating osteoporosis in OVX mice [89].

In summary, SLIT3 regulates bone metabolism and angiogenesis by binding to ROBO receptors. In KOA, aberrantly high SLIT3 expression drives abnormal subchondral type H angiogenesis through the ROBO1/TGF-β1/SMAD pathway, exacerbating joint degeneration. TMJ-OA studies have similarly confirmed that SLIT3 promotes aberrant angiogenesis and nerve invasion, suggesting that this mechanism may have cross-joint commonality. In osteoporosis and fracture healing, SLIT3 exerts bone-protective effects by promoting type H angiogenesis. Therefore, elucidating the dynamic regulation of the SLIT3-ROBO signaling axis at different stages of KOA will provide a theoretical basis for precisely targeting this pathway in KOA treatment.

### 3.3. DLL4/Notch Signaling Axis

The Notch signaling pathway is crucial for bone homeostasis, and its dysfunction is associated with osteoporosis and osteoarthritis [90,91]. Research indicates that the DLL4/Notch signaling axis plays an important regulatory role in bone angiogenesis and can modulate bone metabolism by affecting type H vessel formation [7].

In human OA studies, the tidemark at the osteochondral junction is disrupted by vascular channels, and sensory and sympathetic nerves invade the cartilage along these vascular channels, which is closely associated with OA pain [92]. After the integrity of the osteochondral junction is compromised, aberrant type H vessel proliferation accompanied by sensory nerve invasion along vascular channels into cartilage represents an important mechanism of OA pain [24,93]. The above evidence indicates that the abnormal invasion of blood vessels and sensory nerves in articular cartilage constitutes an important pathological basis for OA pain.

Currently, direct evidence for DLL4/Notch axis regulation of type H vessels mainly derives from non-OA models such as bone repair and osteoporosis, and research in KOA animal models and in vitro experiments remains very limited. In other disease models, the DLL4/Notch axis has been shown to positively regulate type H angiogenesis and bone repair. In a mouse mandibular advancement-induced condylar osteogenesis model, blockade of the DLL4/Notch axis reduced type H vessels, RUNX2^+^ osteoprogenitor cells, and Noggin, impairing MA-induced condylar osteogenesis volume and quality, while also leading to shortened femurs and reduced bone mass [33]. In a mouse rib fracture model, CD90 knockdown inhibited the DLL4/Notch signaling pathway, reduced the number of type H vessels at the injury site, and impaired bone healing [94]. In contrast, the addition of recombinant Dll4 protein (r.Dll4) or overexpression of Notch1 reversed these effects [94]. In a naturally aging osteoporosis mouse model, Bu-Sui-Dan promoted histone acetylation at the DLL4 and Notch1 promoters by upregulating zinc finger E-box-binding homeobox 1, activating the DLL4/Notch1 signaling axis, increasing type H vessel formation, and improving bone mineral density and trabecular microstructure [95]. The above evidence indicates that the DLL4/Notch signaling axis plays a critical role in bone–vessel coupling. Furthermore, in an in vitro cerebral ischemia model, BMSC-derived exosomal miR-148b-3p alleviated microglial activation and neuroinflammation by targeting and inhibiting DLL4 and Notch1 [96], suggesting that overactivation of the DLL4/Notch axis may drive neuroinflammation. Considering the pathological features of vascular and sensory nerve invasion in OA articular cartilage, whether overactivation of the DLL4/Notch axis exacerbates OA pain by promoting coordinated vascular-neural invasion warrants further investigation, although the applicability of this mechanism in KOA remains to be validated.

In summary, the DLL4/Notch axis bidirectionally regulates type H angiogenesis: in the early stages of bone repair, this pathway mediates functional type H angiogenesis and osteogenic coupling. In contrast, in the chronic phase, its overactivation may drive pathological angiogenesis and neuroinflammation. However, the direct role of the DLL4/Notch axis in regulating type H vessels in KOA subchondral bone remains unclear. Future research should focus on elucidating the dynamic changes in this pathway at different stages of KOA and exploring precise therapeutic strategies that moderately inhibit pathological angiogenesis while preserving functional vessels.

### 3.4. HIF/mTOR Signaling Axis

The HIF/mTOR signaling axis plays an important role in regulating type H angiogenesis, cartilage metabolism, and the inflammatory microenvironment. In the KOA process, hypoxia and the inflammation of subchondral bone activate the expression of HIF-1α, which promotes pathological vascular invasion through VEGF effector molecules. This is closely related to the abnormal proliferation of type H vessels.

In human OA cartilage tissue, long noncoding HIF-1α co-activating RNA (LncHIFCAR) and HIF-1α expression are both significantly upregulated [53]. In vitro OA cell models confirmed that inhibition of LncHIFCAR can downregulate the HIF-1α/VEGF axis by blocking the PI3K/AKT/mTOR pathway, thereby alleviating chondrocyte apoptosis and inflammatory responses [53]. In an OA mouse model, Urolithin A reduced the expression of ferroptosis-related markers Malondialdehyde and Fe^2+^ by activating adenosine monophosphate-activated protein kinase (AMPK) and inhibiting the mTOR/HIF-1α pathway in a mouse OA model. It also inhibited the release of inflammatory mediators prostaglandin E2, nitric oxide, and matrix-degrading enzymes MMP1 and MMP3. Urolithin A not only protects cartilage by blocking chondrocyte ferroptosis and the inflammatory response, but also inhibits HIF-1α to reduce the abnormal invasion of pathological type H vessels into subchondral bone, suggesting it as a target for regulating cartilage-blood vessels [54]. Furthermore, HIF-2α also regulates the mTOR pathway. In von Hippel–Lindau cartilage-specific knockout mice, von Hippel–Lindau deletion accelerated age-related and DMM-induced OA progression by upregulating the expression of HIF-2α and phosphorylated mTOR, increasing chondrocyte apoptosis, and reducing autophagy [55]. These findings collectively indicate that excessive activation of the HIF-1α/HIF-2α-mTOR axis plays an important driving role in OA cartilage degeneration.

In in vitro cell experiments, HIF-1α-overexpressing adipose-derived mesenchymal stem cells (ADSCs) enhanced their osteogenic differentiation capacity and promoted tube formation of HUVECs by activating the VEGF/AKT/mTOR signaling pathway [97]. In a Sprague-Dawley rat titanium implant model, HIF-1α overexpression in ADSCs enhanced peri-implant osseointegration [97]. In human periodontal ligament stem cells, hypoxia induced by cobalt chloride promoted HIF-1α expression by upregulating the AKT/mTOR/eukaryotic initiation factor 4E-binding protein 1 pathway, enhancing their osteogenic differentiation and promoting bone regeneration in alveolar bone defects [98]. These studies indicate that the HIF-1α/VEGF/AKT/mTOR axis positively regulates vascular–osteogenesis coupling under hypoxic conditions.

Other non-OA diseases have further elucidated the role of the HIF/mTOR axis in regulating type H vessels and bone metabolism. In a bone defect model, SIRT1 activation promotes FOXO1 phosphorylation/inactivation via PI3K/AKT, enhancing endothelial function, type H angiogenesis, and osteogenesis [31]. However, vascular stability also depends on pericytes. In adult and middle-aged mice, FOXO1 expression in pericytes decreased with age [99]. FOXO1 deletion or inhibition activated the mTOR pathway, driving pericyte-to-myofibroblast transition via PDGFR-β/alpha-Smooth Muscle Actin, impairing type H vessels and causing bone loss, effects that were reversed by rapamycin [99]. Thus, the SIRT1/FOXO1/mTOR/HIF-1α axis plays an important role in type H vessel regulation, and FOXO1 regulation is context-dependent: transient inactivation of FOXO1 in endothelial cells promotes type H angiogenesis, while sustained FOXO1 expression in pericytes maintains vascular integrity. In a rabbit intervertebral disk degeneration model, Erxian decoction delays intervertebral disk degeneration by upregulating p-AKT/p-mTOR/HIF-1α, supporting hypoxic metabolism and reducing inflammation/apoptosis [100]. Unlike HIF-1α, HIF-2α regulates the mTOR pathway in the opposite direction in the bone marrow microenvironment. Wang et al. found that HIF-2α in the bone marrow microenvironment activates the mTOR signaling pathway, promoting adipogenesis of BMSCs and inhibiting their osteogenic differentiation, thereby reducing bone mass [101].

### 3.5. HIF/SLIT Signaling Axis

The regulation of the SLIT family by HIF is subtype-specific and disease context-dependent. In KOA, direct studies on the HIF/SLIT axis in type H vessel regulation are very limited, and the following content is mainly inferred from findings in other disease models.

In non-OA disease models, the role of the HIF-1α/SLIT axis in regulating angiogenesis has been preliminarily revealed. In a rat vascular graft restenosis model, hypoxia-induced HIF-1α upregulation negatively regulated SLIT2 expression, promoting vascular smooth muscle cell migration and proliferation and leading to graft stenosis [102]. Exogenous SLIT2 can reverse this process and maintain vascular patency [102]. In a mouse alveolar bone disuse osteoporosis model, lack of bite force downregulated the expression of HIF-1α in the periodontal ligament, which subsequently inhibited the secretion of SLIT3, decreasing type H angiogenesis and the density of Osx^+^ progenitor cells [103]. Injecting exogenous recombinant SLIT3 could partially reverse type H angiogenesis and bone mass loss [103]. In vitro experiments further confirmed that applying cyclic compression to three-dimensionally cultured periodontal ligament cells promoted type H angiogenesis and osteogenic coupling through the Piezo-type mechanosensitive ion channel component 1/calcium ion/HIF-1α/SLIT3 mechanical signaling axis [103]. These results suggest that the HIF-1α/SLIT3 axis is the key pathway of mechanical–vascular–bone coupling.

However, HIF-2α regulates the SLIT family in the opposite direction to HIF-1α. In human colorectal cancer tissues, SLIT2 is lowly expressed, whereas glypican-1, HIF-1α, and HIF-2α are highly expressed [104]. HIF-2α dominantly downregulates SLIT2 expression, thereby relieving the restriction on glypican-1 and promoting its exosome release, which in turn activates the Wnt signaling pathway to promote tumor cell proliferation [104]. In mice with nucleus pulposus-specific HIF-2α overexpression, sustained HIF-2α activation led to dysregulation of SLIT/ROBO pathway-related genes and accelerated intervertebral disk degeneration [105]. These findings suggest that HIF-2α-dominated SLIT inhibition may be widely involved in tissue homeostasis dysregulation in age-related degenerative diseases.

In summary, the regulation of the HIF/SLIT signaling axis exhibits subtype specificity: HIF-1α typically positively regulates SLIT3, promoting angiogenesis and bone repair. In contrast, HIF-2α inhibits SLIT2, driving pathological tissue remodeling. It is speculated that local hypoxia or inflammation in early KOA may repair bone vessels by activating the HIF-1α/SLIT3 axis, and with disease progression, HIF-2α-dominated activation may inhibit SLIT expression and further disrupt the bone–vascular coupling balance, thereby exacerbating subchondral bone destruction. However, the above speculation awaits experimental validation in KOA animal models and human OA samples. Therefore, targeting the HIF/SLIT signaling axis and restoring the HIF-1α/HIF-2α-SLIT balance may represent a new strategy for delaying KOA progression.

### 3.6. LOX-1/HIF-1α/SLC7A11 Signaling Axis

In recent years, SLC7A11 has been found to be a key transporter regulating ferroptosis, to play an important role in maintaining the function of type H vascular endothelial cells (THVECs), and to participate in the OA pathological process through a variety of mechanisms [106,107]. As a functional subunit of the Xc^−^ system, SLC7A11 is involved in the synthesis of glutathione, which assists glutathione peroxidase 4 (GPX4) in clearing lipid peroxides and inhibiting ferroptosis.

In a DMM-induced rat OA model, α-ketoglutarate reduced reactive oxygen species and malondialdehyde levels, increased superoxide dismutase and GSH levels, decreased ferrous ion accumulation, preserved mitochondrial membrane potential, inhibited chondrocyte ferroptosis and matrix degradation, and alleviated joint degeneration in OA rats by activating the ETS translocation variant 4/SLC7A11/GPX4 signaling pathway [106]. In human OA chondrocytes, the expression of ferroptosis inhibitor genes, such as *SLC7A11* and *GPX4,* is downregulated, leading to increased ferroptosis [108]. Further bioinformatics analysis identified GPX4 and SLC7A11 as target genes with therapeutic potential for OA. The natural compound resveratrol was found to upregulate these genes, inhibit ferroptosis, and thereby protect chondrocytes [109]. The above evidence indicates that inhibition of the SLC7A11/GPX4 axis is closely associated with ferroptosis in OA chondrocytes.

In other bone disease models, the role of the LOX-1/HIF-1α/SLC7A11 axis in type H vascular endothelial cell ferroptosis and bone metabolism regulation has been preliminarily revealed. In in vitro experiments, high glucose-treated rat THVECs exhibited elevated LOX-1 expression, which inhibited the HIF-1α/SLC7A11 pathway, leading to increased reactive oxygen species, malondialdehyde, and ferrous ion levels, decreased GSH levels, and promoted THVEC ferroptosis [107]. LOX-1 silencing can restore the activity of the HIF-1α/SLC7A11 signaling axis and upregulate the expression of GPX4, effectively preventing the hyperglucose-induced ferroptosis of THVEC, thereby restoring the integrity and function of type H vessels [107]. In in vivo experiments, LOX-1 knockdown in db/db diabetic mice improved bone quality and promoted type H angiogenesis, further validating that LOX-1 regulates bone metabolism and type H vessel homeostasis [110]. These studies indicate that LOX-1 is a key regulator linking metabolic abnormalities to bone-type H angiogenesis, and that it promotes type H vascular endothelial cell ferroptosis and disrupts bone–vascular coupling by inhibiting the HIF-1α/SLC7A11 axis.

In summary, the LOX-1/HIF-1α/SLC7A11 signaling axis plays an important regulatory role in type H vascular endothelial cell ferroptosis and bone metabolism. In OA, downregulation of SLC7A11 and GPX4 expression has been confirmed. However, the direct role of this axis in type H vessels of KOA subchondral bone remains to be validated. We speculate that this axis may exhibit opposite regulation in different skeletal sites of KOA. In the diaphysis, LOX-1 upregulation may inhibit the HIF-1α/SLC7A11 axis, promote type H vascular endothelial cell ferroptosis, and impair bone repair. In contrast, in subchondral bone, excessive activation of the HIF-1α/SLC7A11 axis may drive pathological type H vessel proliferation, exacerbating joint degeneration. Therefore, precisely regulating this signaling axis at different sites, by promoting functional angiogenesis in the diaphysis while inhibiting pathological vessel invasion in subchondral bone, may represent a new direction for KOA treatment, but this awaits experimental validation.

Despite the substantial progress in elucidating the regulatory mechanisms of type H angiogenesis in KOA, several conflicting findings, methodological limitations, and model-specific differences warrant careful attention.

Regarding conflicting findings, the dual role of HIF-1α represents a prominent paradox. HIF-1α promotes functional angiogenesis and bone repair in the diaphyseal region, yet its excessive accumulation in subchondral bone drives pathological vascular invasion and cartilage degeneration. Similarly, SLIT3 exerts bone-protective effects by promoting type H vessel formation in osteoporosis models, whereas its upregulation in KOA subchondral bone exacerbates pathological angiogenesis and sensory nerve invasion. The DLL4/Notch axis may also exhibit bidirectional regulation, mediating functional angiogenesis–osteogenesis coupling during early bone repair, while its overactivation may drive pathological angiogenesis and neuroinflammation. Furthermore, HIF-3α presents a notable contradiction: its high expression in healthy cartilage negatively correlates with hypertrophic markers, yet miR-210-mediated inhibition of HIF-3α promotes chondrocyte proliferation and extracellular matrix deposition, improving OA. These discrepancies may be partially attributed to differences in detection levels, i.e., total mRNA versus protein expression, and the functional heterogeneity of splice variants.

Regarding methodological limitations, several shortcomings exist in current research. First, most studies rely on CD31/EMCN immunofluorescence staining with semi-quantitative analysis, which cannot fully capture the three-dimensional architecture of the vasculature. Second, human OA specimens are predominantly obtained from end-stage patients undergoing joint replacement surgery, precluding the assessment of early and mid-stage pathological changes. Third, the majority of studies employ single time-point observations, failing to capture the dynamic evolution of type H vessels during KOA progression. Fourth, key signaling axes, including HIF/SLIT and LOX-1/HIF-1α/SLC7A11, have been primarily validated in non-OA disease models such as osteoporosis, vascular restenosis, and diabetic retinopathy, and their direct relevance to KOA awaits confirmation.

Regarding differences between experimental models, surgical OA models (e.g., DMM and ACLT) differ in their induction mechanisms. DMM mimics chronic joint instability, whereas ACLT induces acute joint instability, which may lead to variations in the time window, severity, and molecular mechanisms of type H vessel changes. Chemically induced models (e.g., papain and monosodium iodoacetate) may activate distinct pathological pathways. Furthermore, the baseline levels and responsiveness of type H vessels vary considerably across species (mouse, rat, human), yet multi-species cross-validation remains largely absent. The anatomical site of investigation is also a critical factor, as type H vessels in the diaphysis, subchondral bone, mandibular condyle, and intervertebral disk exhibit distinct regulatory mechanisms and functional outcomes.

Therefore, future studies should prioritize multi-timepoint, multi-model experimental designs with systematic cross-validation across species and anatomical sites, so as to resolve current contradictions and establish a comprehensive understanding of the spatiotemporal regulation of type H vessels in KOA.

## 4. Pharmacological Modulation of Type H Vessel Homeostasis in KOA

Given the marked spatial specificity of type H vessel regulation in KOA, where physiological vessels in the diaphyseal region promote bone repair, whereas pathological vessels in the subchondral bone drive joint destruction, novel therapeutic strategies should aim to simultaneously promote diaphyseal repair and suppress subchondral pathology. Accordingly, therapeutic strategies targeting type H vessels are progressively shifting toward multidimensional and precise interventions (Table 2).

### 4.1. Small Molecule Signaling Modulators: Spatiotemporal Regulation of Type H Angiogenesis

#### 4.1.1. Inhibition of Type H Vessels in Subchondral Bone Lesions Can Delay OA Progression

Unlike the diaphysis, the abnormal proliferation of type H vessels in subchondral bone is an important pathological factor driving OA progression [18,50,111]. In human OA studies, Li et al. further confirmed that the high expression of SLIT3 in subchondral bones of OA patients was positively correlated with VEGF expression [50]. In vitro experiments confirmed that osteoblast-derived SLIT3 combined with ROBO1 to activate the transforming growth factor beta1 (TGF-β1)/Sma- and Mad-related proteins (SMAD) pathway, the migration and angiogenesis of endothelial progenitor cells (EPCs) were enhanced by up-regulating the expression of VEGF, CD31, and EMCN [50]. In an ACLT-induced OA mouse model, intra-articular injection of sh-SLIT3 lentivirus (knocking down SLIT3) reduced OARSI scores, and decreased the number of CD31^+^EMCN^+^ endothelial cells [50]. Therefore, inhibiting the SLIT3/Robo1 signaling is a promising new strategy. Although this approach reduces type H vessel numbers, the regulation of Notch signaling needs to be precise, as blocking DLL4/Notch may also impair normal condylar osteogenesis [33]. Another study found that osteoblast lineage cells in the subchondral bone of OA patients highly secrete prostaglandin E2 [112]. In an OA mouse model, it was further confirmed that the prostaglandin E2/prostaglandin E receptor 4 axis in osteoclasts upregulates PDGF-BB through the guanine nucleotide-binding protein G(s) subunit alpha/PI3K/AKT/MAPK pathway, promoting aberrant type H vessel proliferation and sensory nerve innervation in subchondral bone and exacerbating pain. Notably, osteoclast-specific knockout of prostaglandin E receptor 4 or treatment with a novel Prostaglandin E receptor 4 antagonist both reduced aberrant type H angiogenesis and alleviated OA [112].

In animal KOA models, multiple studies have confirmed the therapeutic potential of inhibiting aberrant type H angiogenesis through different signaling axes. Cui et al. used ACLT to establish a mouse model of OA and found that injecting AAV9 (specifically inhibiting endothelial PDGFR-β) into subchondral bone inhibited the talin1/FAK signaling axis to block type H vessel abnormal proliferation in subchondral bone and alleviate the degree of subchondral bone and cartilage lesions. Thus, the pathological process of OA was effectively interrupted [9]. Li et al. found that the Stat3 inhibitor Stattic dose-dependently inhibited type H angiogenesis in the subchondral bone of DMM mice, with almost no type H vessels in the subchondral bone of the 20 mg/kg group, similar to the normal group [111]. Stattic also dose-dependently downregulated angiogenic factors such as VEGF and angiopoietin-2, and reduced the expression of inflammatory factors including IL-6 and tumor necrosis factor-α (TNF-α) [111]. Lin et al. demonstrated in an ACLT combined with DMM-induced OA mouse model that osteopontin (OPN) expression was significantly increased in subchondral bone, accelerating subchondral bone turnover and remodeling by activating the PI3K/AKT signaling pathway, promoting type H angiogenesis, and leading to articular cartilage degeneration [113]. Injection of an OPN neutralizing antibody in OA mice reduced type H angiogenesis in subchondral bone [113].

In cell/molecular mechanism studies, Liu et al. identified an Angptl7^+^ AngC-2 subpopulation in the femoral condyles of KOA mice through single-cell transcriptome sequencing, which interacts with endothelial cells through the FGF2-FGFR2 signaling pathway to promote subchondral type H angiogenesis [8]. In vitro experiments confirmed that Angptl7 overexpression aggravated chondrocyte degeneration, reduced collagen type II alpha 1 chain and aggrecan expression, and increased ADAMTS5 and MMP13 expression [8]. Co-culture of Angptl7^+^ chondrocytes with human umbilical vein endothelial cells (HUVECs) enhanced tube formation, an effect amplified by FGF2 and blocked by the FGFR2 inhibitor Alofanib, indicating that Angptl7 acts through FGF2-FGFR2 signaling [8].

In conclusion, the future treatment of KOA should be based on the specific spatial regulation of signaling molecules. In the subchondral bone lesion area, pathological angiogenesis can be reduced by inhibiting the expression of SLIT3, E-prostanoid 4 receptor, PDGF-β, Stat3, OPN, Angptl7, and FGF2. In the diaphysis area, PDGFR-β activation, RKIP knockout, LOX-1 silencing, and other strategies can be used to promote functional type H angiogenesis. This dual mode of inhibiting pathology and promoting physiology provides an important direction for the development of precise targeted therapies for OA.

#### 4.1.2. Promoting Type H Angiogenesis in the Diaphyseal Region to Maintain Bone Homeostasis

Promoting type H angiogenesis in the diaphysis has potential therapeutic value in bone regeneration and homeostasis maintenance. However, current related strategies have mainly been validated in osteoporosis and diabetic bone disease models, with very limited direct evidence in KOA.

In other disease models, several strategies have demonstrated significant potential for promoting type H angiogenesis and bone repair. In osteoporosis models, PDGFR-β expression is significantly reduced in the bone tissue of both osteoporosis patients and OVX mice [114]. Endothelium-specific enhancement of PDGFR-β can induce endothelial cell to differentiate into H-type phenotype through the p21-activated kinase 1/Notch1/Notch Intracellular Domain signaling cascade, promote vascularized bone regeneration, and reverse bone loss in OVX mice [114]. Knocking out Raf kinase inhibitor protein (RKIP) can inhibit the differentiation of macrophages into osteoclasts in the bone microenvironment and stabilize the expression of HIF-1α to promote the differentiation of macrophages into pro-angiogenic subsets. Thus, the ratio of type H endothelial cells (CD31^hi^EMCN^hi^)/total endothelial cells in mice was increased, the number of osteoblasts (Osteocalcin^+^) was increased, and bone loss in OVX mice was effectively reversed [115]. In diabetic bone disease models, LOX-1 expression is elevated in high glucose-treated THVECs, promoting ferroptosis by inhibiting the HIF-1α/SLC7A11/GPX4 axis [107]. Silencing LOX-1 can restore type H angiogenesis and osteogenesis–vascular coupling [107]. The application of the above strategies in the KOA diaphysis awaits further validation. However, given that age- or metabolism-related bone loss also occurs in KOA, promoting functional type H angiogenesis may provide new insights for restoring bone–vascular coupling balance.

### 4.2. Biomaterial-Mediated Reconstruction of the Vascular–Osteogenic Coupling Niche

Due to the limited self-repair ability of bone tissue, new bone tissue engineering materials, especially 3D-printed scaffolds, are being developed to regulate type H angiogenesis in the bone microenvironment to promote bone repair. This concept also provides a new strategy for reconstructing subchondral bone in KOA [126]. However, current relevant evidence mainly derives from bone defect and osteoporosis models, and direct validation experiments in KOA remain very limited. Yan et al. designed a 3D-printed polycaprolactone (PCL) scaffold modified with EPLQLKM and SVVYGLR peptides (PCL-SE), which could recruit BMSCs and EPCs, respectively. The study further confirmed that PCL-SE promoted EPCs to differentiate into CD31^+^EMCN^+^ type H vessels and upregulated the expression of osteogenesis-related genes in BMSCs, leading to vessel and bone regeneration [116]. This vascular–osteogenesis coupling between BMSCs and type H vessels was also demonstrated in the femoral condyle bone defect osteoporotic rat model [116]. The molecular mechanism of vascularization and osteogenesis of a tannic acid-modified biomimetic mineralized decellularized adipose tissue (TA@mDAT) scaffold developed by Wang et al. may involve increasing the formation of type H vessels and the upregulation of the DLL4-Notch1 pathway, thereby promoting bone regeneration [127]. These studies provide an experimental basis for using bone tissue engineering materials to treat OA.

**Figure 4:**
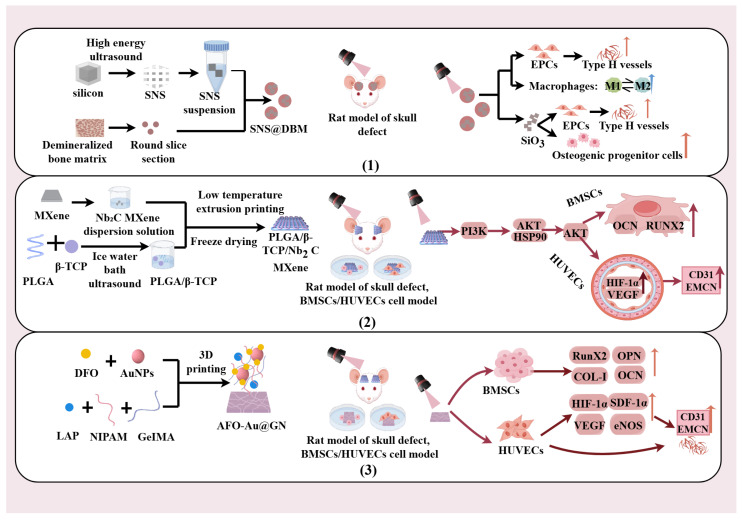
Targeting therapeutic strategies for type H vessels to enhance vessel-bone coupling. This schematic illustrates three representative biomaterial-based strategies for promoting bone regeneration through the enhancement of type H vessel-mediated vascular–osteogenic coupling. (**1**) SNS@DBM intelligent bone scaffold. The fabrication process of the SNS@DBM smart bone scaffold is shown. In a rat calvarial defect model, the SNS@DBM scaffold enhances osteogenesis, promotes CD31^+^EMCN^+^ type H angiogenesis, and induces M2 macrophage polarization. (**2**) 3D-printed PTMN hydrogel scaffold. The preparation of the PTMN hydrogel scaffold is schematically illustrated. In a rat calvarial defect model and BMSC/HUVEC co-culture systems, the PTMN scaffold enhances CD31^+^EMCN^+^ type H vessel formation and new bone regeneration by activating the HIF-1α/STAT3/VEGF signaling axis and the PI3K-AKT signaling pathway, synergistically achieving angiogenesis–osteogenesis coupling. (**3**) 3D-printed DFO-Au@GN hydrogel scaffold. The synthesis route of the DFO-Au@GN hydrogel scaffold is shown. In a rat calvarial defect model and BMSC/HUVEC co-culture systems, the DFO-Au@GN scaffold activates the HIF-1α signaling pathway to promote type H angiogenesis and enhance the osteogenic differentiation of bone marrow mesenchymal stem cells, thereby achieving angiogenesis–osteogenesis coupling. Abbreviations: AKT, AKT serine/threonine kinase; AuNPs, gold nanoparticles; BMSCs, bone marrow mesenchymal stem cells; CD31, platelet endothelial cell adhesion molecule-1; COL-I, type I collagen; DBM, demineralized bone matrix; DFO, deferoxamine; eNOS, endothelial nitric oxide synthase; EMCN, endomucin; EPCs, endothelial progenitor cells; GelMA, gelatin methacryloyl; HIF-1α, hypoxia-inducible factor 1α; HSP90, heat shock protein 90; HUVECs, human umbilical vein endothelial cells; LAP, lithium phenyl-2,4,6-trimethylbenzoylphosphinate; M1, classically activated macrophage; M2, alternatively activated macrophage; MXene (Nb_2_C), niobium carbide MXene; NIPAM, N-isopropylacrylamide; OCN, osteocalcin; OPN, osteopontin; PI3K, phosphoinositide 3-kinase; PLGA, poly(lactic-co-glycolic acid); RUNX2, runt-related transcription factor 2; SDF-1α, stromal cell-derived factor 1α; SiO_3_^2−^, silicate ion; SNS, silicon nanosheets; VEGF, vascular endothelial growth factor; β-TCP, beta-tricalcium phosphate. This illustration was created using Figdraw (www.figdraw.com, accessed on 19 July 2026) with copyright code OTSRP355ed.

Cheng et al. constructed a multifunctional “thermoswitch” intelligent bone scaffold, demineralized bone matrix modified by siliconene nanosheets (SNS@DBM). This scaffold could effectively prevent tumor recurrence and promote bone regeneration through the intense photothermal removal of residual tumors and the moderate thermal enhancement of osteogenesis, CD31^+^EMCN^+^ type H angiogenesis, and M2-type macrophage polarization. The technique provides a new method for the precise treatment of complex bone diseases [117] (Figure 4). A 3D-printed hydrogel scaffold (PTMN scaffold) loaded with nanomaterials (Nb_2_C MXene) effectively promoted vascular basement membrane degradation by upregulating the expression of MMP-1, MMP-3, and MMP-10 through mild hyperthermia, and activated the HIF-1α/STAT3/VEGF signaling axis in HUVECs to enhance angiogenesis. This scaffold also activated the PI3K-AKT signaling pathway in BMSCs, significantly improving repair efficiency in a rat bone defect model. The proportion of CD31^+^EMCN^+^ type H vessels was markedly increased and highly coincident with the location of new bone, indicating that the PTMN scaffold activated angiogenesis through HIF-1 and subsequently upregulated the PI3K-AKT pathway to activate osteogenesis, synergistically achieving vascular–osteogenesis coupling [118] (Figure 4). Another innovative multifunctional and smart scaffold is the near-infrared antibacterial 3D-printed hydrogel scaffold (DFO-Au@GN), which is composed of desferrioxamine (DFO), gold nanoparticles (AuNPs), gelatin methacrylate (GelMA), and N-isopropylacrylamide (NIPAM). This scaffold promoted endothelial cells to form CD31^+^EMCN^+^ type H vessels in vitro and enhanced the osteogenic differentiation ability of BMSCs, thereby improving the repair efficiency of rat skull defects [119] (Figure 4). Notably, a bone morphogenetic protein-2 (BMP-2)/sulfated chitosan (SCS)/calcium phosphate cement (CPC) composite scaffold inhibited BMP-2-induced osteoclast overactivation, reduced MMP-9 expression, restored osteogenic/chondrogenic differentiation of vertebral skeletal stem cells, promoted type H vessels and new bone formation, and increased the spinal fusion success rate from 16.7% to 83.3% in aged mice [120]. This indicates that the composite scaffold can alleviate age-related bone microenvironment dysgenesis and reconstruct the bone microenvironment by restoring vascular–osteogenesis coupling, providing a potential strategy for OA treatment. In summary, bone tissue materials and intelligent targeting technology can promote bone repair by accurately regulating the coupling of angiogenesis and osteogenesis, providing new therapeutic ideas for KOA. However, their applicability in KOA subchondral bone reconstruction awaits targeted validation.

### 4.3. Pharmacological Regulation of Type H Vessel Homeostasis

#### 4.3.1. Natural Products and Biomaterials Mediating Bidirectional Regulation of Type H Vessels: From Molecular Mechanisms to Targeted Therapeutic Strategies

Traditional Chinese Medicine offers multi-target regulation with significant potential for chronic bone diseases. However, type H vessels exhibit spatial specificity. In KOA animal models, several natural products and biomaterials have demonstrated potential in inhibiting pathological type H angiogenesis in subchondral bone. Soybean isoflavone effectively ameliorates OA by activating tuberous sclerosis complex 1 (TSC1) and suppressing the mTORC1/VEGF axis, thereby reducing pathological type H angiogenesis in the subchondral bone of OA rats [121]. Similarly, dibutyl phthalate from Panax notoginseng inhibits excessive osteoclast activation and type H vessel invasion in the subchondral bone of ACLT-induced OA mice [38]. Advanced delivery systems, Zuo et al. used high-permeability micro/nano hydrogel microspheres (SCT-HA), composed of selenium-doped carbon quantum dots, triphenylphosphine, and modified hyaluronic acid to precisely target drug delivery to subchondral CD31^hi^EMCN^hi^ vessels in an ACLT-induced OA mouse model, normalizing the number of aberrantly proliferating type H vessels and correcting imbalanced subchondral bone remodeling [122].

In non-OA models such as osteoporosis, some natural products can promote type H angiogenesis and bone repair, but their application in the KOA diaphysis awaits further validation. In an OVX-induced osteoporosis mouse model, daidzein activate the EGFR/AKT/PI3K axis to enhance type H angiogenesis and bone formation [17]. YSTG upregulated SLIT3 expression and increased the number of type H endothelial cells in the diaphysis [88]. In in vitro cell co-culture and in vivo bone defect models, the combination of natural plant-derived polyphenolic tannic acid with LL-37 induced macrophage polarization toward the M2 phenotype and significantly promoted type H angiogenesis and bone regeneration, achieving efficient tissue repair [123].

In summary, the integration of the multi-target pharmacological activities of natural products with advanced drug delivery systems can achieve spatiotemporal precision in regulating type H vessels—suppressing pathological angiogenesis in subchondral bone while promoting functional vascular regeneration in the diaphysis. The therapeutic potential of bioactive metabolites from traditional Chinese botanical drugs targeting type H vessels has attracted widespread attention [128], establishing a novel pharmacotherapeutic paradigm for KOA with promising clinical translational prospects.

#### 4.3.2. Pharmacological Intervention Targeting the Senescent Microenvironment: From Senolytic Clearance to Precision Regulation of Angiogenesis–Osteogenesis Coupling

Cellular senescence exhibits a dual pharmacological character within the bone microenvironment. Currently, the interaction between the aging microenvironment and type H angiogenesis has been mainly revealed in models of spinal degeneration and bone defects, but research in KOA is still in its infancy.

In other disease models, multiple studies reveal the complexity of senescent cell regulation of type H angiogenesis. In lumbar instability and aging mouse models, the number of senescent osteoclasts (SnOCs) is significantly increased, promoting aberrant sensory nerve innervation and type H angiogenesis, thereby exacerbating pain. Navitoclax (ABT263) alleviates spinal pain by eliminating SnOCs in the vertebral endplate. This treatment reduces endplate porosity and inhibits CD31^hi^EMCN^hi^ type H angiogenesis, subsequently decreasing sensory nerve (calcitonin gene-related peptide^+^ and protein gene product 9.5^+^) ingrowth [16]. Endothelial homeostasis, vital for type H vessel formation, is regulated by transient receptor potential melastatin 7 (TRPM7). In endothelial-specific TRPM7 knockout mice and a hindlimb ischemia model, TRPM7 knockout disrupts lactate metabolism and induces vascular senescence via upregulation of cyclin-dependent kinase inhibitor 1A (p21), leading to microvascular rarefaction [124]. This can be reversed by p21 inhibition or lactate supplementation [124]. Conversely, some senescent cells possess pro-regenerative functions. Zhao et al. identify “senescent-like macrophages” that promote bone regeneration. In a mouse calvarial defect model, 6-Hydroxydopamine inhibition of sympathetic nerves increased type H vessels and these beneficial macrophages, an effect abolished by ABT263 [125].

The above findings suggest that SnOCs-driven pathological neurovascular invasion represents a target for pharmacological clearance, while the TRPM7 pathway and pro-regenerative senescent macrophages serve as pivotal avenues for restoring bone tissue repair potential. Future therapeutic strategies for KOA should focus on developing spatiotemporally selective senolytics or microenvironment modulators that not only eliminate pathogenic senescent cells but also activate endogenous pro-regenerative mechanisms, thereby achieving homeostatic remodeling of angiogenesis–osteogenesis coupling. However, the applicability of the above strategies in the KOA subchondral bone microenvironment awaits targeted validation.

## 5. Challenges and Prospects

**Figure 5:**
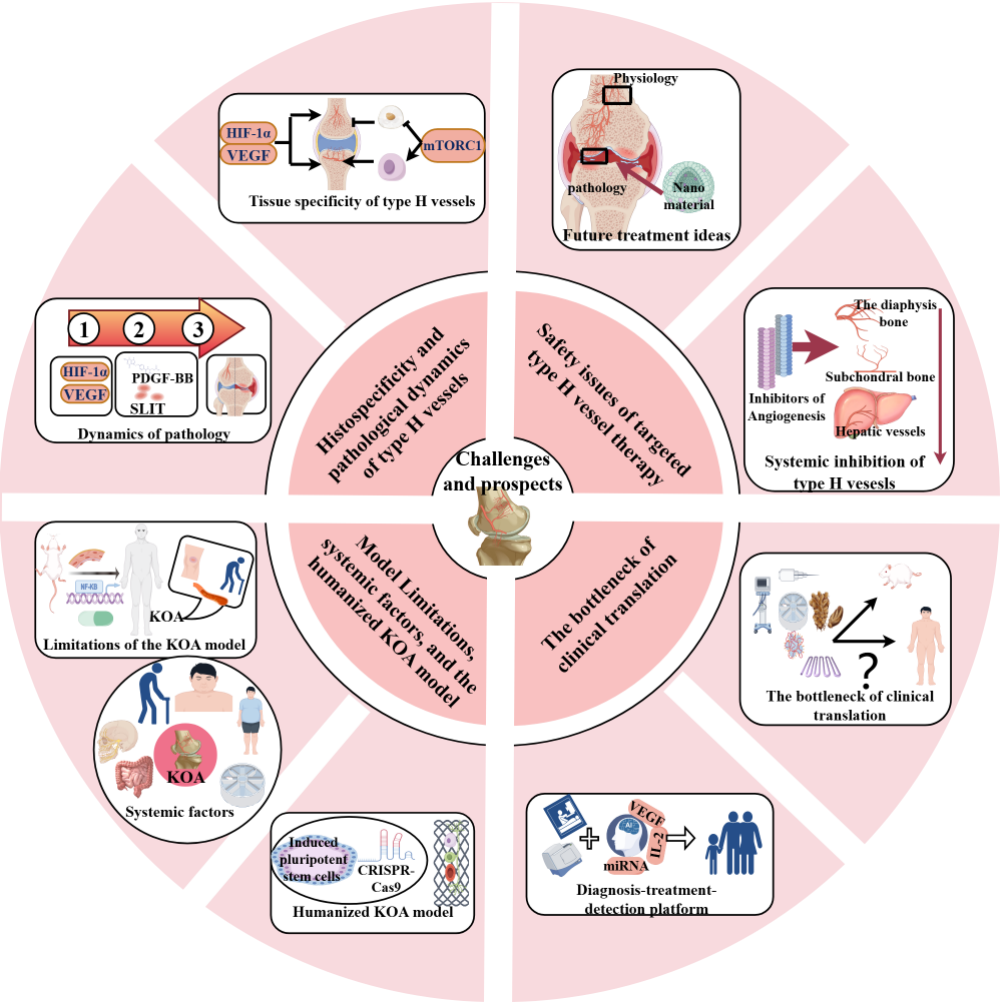
Challenges and prospects for targeting type H vessels in the treatment of knee osteoarthritis. This figure summarizes the four major challenges in translating type H vessel research into clinical applications: (1) tissue specificity and pathological dynamics of type H vessels; (2) safety concerns of targeted therapy; (3) model limitations, systemic factors, and humanized KOA models; (4) bottlenecks in clinical translation and multimodal platform development. Abbreviations: CRISPR-Cas9, clustered regularly interspaced short palindromic repeats–CRISPR-associated protein 9; HIF-1α, hypoxia-inducible factor 1-alpha; IL-2, interleukin-2; KOA, knee osteoarthritis; miRNA, microRNA; mTORC1, mechanistic target of rapamycin complex 1; PDGF-BB, platelet-derived growth factor BB; SLIT, slit guidance ligand; VEGF, vascular endothelial growth factor. This illustration was created using Figdraw (www.figdraw.com, accessed on 19 July 2026) with copyright code ROUUU353a5.

Although progress has been made in studying the ability of type H vessels to regulate KOA pathogenesis, many key challenges remain, including the dual role of type H vessels in different bone regions, the temporal and spatial heterogeneity of the signal axis regulating type H vessels, the limitations of animal models, and the safety considerations of targeted therapy. Therefore, before extending to clinical application, we should focus on solving the following problems in related basic research (Figure 5).

First, the tissue specificity and pathological dynamics of type H vessels are a major obstacle to the precise treatment of KOA. In KOA, type H vessels promote bone repair in the diaphysis region, while in subchondral bone, they lead to osteophyte formation, bone remodeling imbalance, and abnormal nerve invasion, which aggravate disease progression. Due to these dual characteristics, therapeutic methods targeting a certain area may have limitations, such as poor efficacy and safety [129]. For example, HIF-1α/VEGF promotes functional angiogenesis in the diaphysis region but induces pathological type H vessel invasion in subchondral bone. Similarly, mTORC1 activation was shown to promote type H angiogenesis in chondrocytes but inhibit this process in osteoblasts [11]. The tissue specificity of type H vessels was further confirmed in the calvarial defect model [130]. A large number of typical type H vessels (CD31^+^EMCN^+^) were observed in the cortical bone marrow cavity of the skull, which were closely adjacent to clusters of osteoblasts. On the surface of the upper periosteum and dura mater, the vessels were mainly composed of ordinary capillaries, and the content of type H vessels was very low [130]. This suggests that the therapeutic strategy of targeting type H vessels should consider its anatomical location specificity. The regulation of type H vessels is also stage-dependent. HIF-1/VEGF is dominant in the early stage, PDGF-BB, SLIT, and other factors play a prominent role in the middle stage, and the late stage is accompanied by aggravated bone sclerosis and sparse functional vessels [22,131,132]. Therefore, future therapeutic strategies should focus on developing spatiotemporally precise delivery systems [9,133] In subchondral bone lesions, AAV vectors or nanomaterial-loaded hydrogels targeting CD31^hi^EMCN^hi^ type H vessels can be designed to achieve precise drug release through local injection, selectively inhibiting pathological signaling axes such as PDGFR-β/talin1/FAK and Slit3/ROBO1, thereby blocking aberrant angiogenesis and sensory nerve invasion. Meanwhile, in the diaphyseal region, biomaterials loaded with SIRT1 agonists can be employed to promote functional type H vessels formation and angiogenesis–osteogenesis coupling. Furthermore, multi-omics technologies, including single-cell transcriptome sequencing and spatial metabolomics, should be integrated to systematically map the dynamic molecular atlas of type H endothelial cells across different KOA stages (early, middle, and end-stage) and anatomical regions (subchondral bone, diaphysis, synovium, and meniscus). This will facilitate the identification of tissue-specific biomarkers and druggable targets, providing a guiding roadmap for differential precision therapy aimed at inhibiting pathological regions while promoting physiological regions.

Second, the safety of targeting type H vessels represents a critical bottleneck limiting clinical translation. Type H vessels not only drive the pathological progression of KOA subchondral bone [9], but are also essential for maintaining physiological bone repair and fracture healing in the metaphysis of long bones [29,31]. Systemic inhibition of type H angiogenesis, such as through the use of VEGF inhibitors or mTOR inhibitors, may reduce pathological vascular proliferation in subchondral bone while simultaneously disrupting normal vascular–osteogenesis coupling in the diaphysis, leading to bone loss. In addition, angiogenesis inhibitors may produce off-target effects in multiple organ systems. For example, systemic blockade of the Notch signaling pathway not only affects bone vessels but may also impair normal vascular function in other organs [33]. Given the opposing functional tendencies of type H vessels in KOA subchondral bone and the diaphysis, future therapies must distinguish between pathological and physiological roles. In terms of precise delivery, on the one hand, nanocarriers or AAV vectors surface-modified with CD31 or EMCN antibodies can be developed to achieve active targeting of subchondral type H vessels, enabling the precise delivery of anti-angiogenic drugs (e.g., Stattic) to the lesion area while minimizing off-target effects on the diaphyseal region. On the other hand, local intervention strategies, such as ultrasound-guided intra-articular injection and percutaneous microneedle array drug delivery, should be further explored to enhance local drug concentration and bioavailability, thereby reducing the risk of bone loss associated with systemic exposure. Concurrently, a multidimensional efficacy and safety monitoring system incorporating serum bone turnover markers and vascular imaging should be established to assess the effects of treatment on diaphyseal bone mass and fracture healing capacity in real time, enabling precise control of the therapeutic window.

Third, the constructed KOA research model has great limitations in simulating the human pathological environment. Although mouse models of ACLT or DMM have been widely used for mechanism exploration, it is difficult for these models to cover the multifactorial characteristics of the elderly population, such as chronic low-grade inflammation, systemic metabolic disorders (such as diabetes), cell senescence accumulation, and neurovascular cooperative invasion in subchondral bone [24,134,135,136]. Most importantly, the phenotypes and regulatory networks of type H vessels in human subchondral bone have not been verified by clinical trials. However, many studies using mouse models ignore the significant species differences between humans and rodents in endothelial cell subtypes, signaling pathway conservation, and drug responsiveness [137,138]. Therefore, it is urgent to construct a humanized KOA model that is more suitable for translation in clinical practice. On the one hand, osteochondral organoids can be constructed based on patient-derived induced pluripotent stem cells, which can better simulate the bone-cartilage-vascular-nerve microenvironment in KOA [139,140]. Combined with gene-editing platforms (such as CRISPR-Cas9), OA risk genes (such as *ERG*, *PIK3R1*, and *WNT3*) can be introduced to establish a disease model with genetic background specificity [141,142]. On the other hand, by combining human primary type H endothelial cells, AngC-2 chondrocytes and sensory neurons in a bionic 3D scaffold, a more comprehensive 3D system can be constructed to further analyze the coupling mechanism of cartilage-vascular-nerve and provide more reliable experimental verification for targeted therapy [8,143]. In addition, the regulation of type H vessels by systemic factors and multi-system interaction cannot be ignored. Studies have shown that systemic states, such as obesity, diabetes, and aging, are independent risk factors for the occurrence of KOA [144,145,146], which may involve intestinal microbiota (such as the gut–articular axis), or the neuroendocrine system to regulate type H angiogenesis in the KOA microenvironment. For example, HIF-2α not only drives type H angiogenesis, but is also regulated by gut microbiota metabolites [44,45]. At the same time, senescent osteoclasts are also an important factor driving pathological type H vessels–nerve coordination to invade subchondral bone. In the future, multi-system synergistic regulation may be explored to restore type H vessel homeostasis in KOA, thereby opening new therapeutic avenues. Regarding the gut–joint axis, supplementation with probiotics (e.g., *Latilactobacillus sakei* LB-P12) or specific dietary fibers may modulate gut microbiota metabolites, inhibit the NF-κB/HIF-2α signaling pathway, and reduce pathological type H vessel formation in subchondral bone. In terms of neuromodulation, selective blockade of sympathetic nerve signals may suppress aberrant neurovascular co-invasion and alleviate OA pain. With respect to oxygen metabolism regulation, hyperbaric oxygen therapy improves the local oxygen environment via the PHD2/HIF-1α pathway [41], and swimming exercise inhibits aberrant type H vessel proliferation in subchondral bone by downregulating the HIF-1α/VEGF axis [40], suggesting that improving systemic oxygen metabolism and the joint mechanical microenvironment represents a feasible adjunctive strategy. Furthermore, the selective elimination of senescent osteoclasts (e.g., using Navitoclax) can block SnOC-mediated pathological type H vessel–nerve co-invasion [19], offering a new direction for the application of anti-aging therapies in KOA. These multi-system, multi-target synergistic intervention strategies hold promise for overcoming the limitations of single-pathway inhibition and achieving holistic treatment of KOA.

Finally, translating treatment strategies based on type H vessels into clinical practice remains challenging. Physical therapy, such as hyperbaric oxygen, swimming, fire needling, and traditional Chinese medicine, has shown great potential in regulating type H angiogenesis in animal models. However, problems remain, such as unclear mechanisms, low bioavailability, and large individual differences [147,148]. At the same time, although new intelligent biomaterials (such as PTMN scaffolds) can achieve accurate delivery and improve repair rates, their retention, biocompatibility, and safety in human joints are still unclear [149,150,151]. Therefore, a multi-modal integrated diagnosis-treatment-monitoring platform should be developed. On the one hand, CD31^+^EMCN^+^ signals are used as an imaging marker of type H vessels, and dynamic changes in type H vessels can be monitored using positron emission tomography and magnetic resonance imaging technology. On the other hand, artificial intelligence technology can be used to integrate the patient’s serum VEGF, inflammatory factor profile (IL-2/4/6), specific miRNA (miR-210/miR-29cb2), and other multi-dimensional data to construct an effective, personalized predictive model. It can be used to guide the timing and dosage of HIF, SLIT, mTOR, and other important signaling axis inhibitors and realize the transformation from the multi-range inhibition of blood vessels to the fine remodeling of the vascular ecology, and bring long-lasting and safe joint function protection to KOA patients.

## 6. Conclusions

In conclusion, this review highlights the interplay between the tissue specificity of type H angiogenesis and KOA pathophysiology and disease progression, as well as the intrinsic regulatory mechanisms of type H angiogenesis. We systematically reviewed the current therapeutic strategies targeting type H angiogenesis in promoting bone regeneration and maintaining bone homeostasis. The major challenges and future research directions are outlined to promote the translation of basic research findings into clinical applications. These include addressing the tissue specificity and pathological dynamics of type H vessels, establishing safety assessment and optimization systems for targeted therapy, developing disease models relevant to humans, exploring combinatorial pharmacotherapy, and breaking through the technical bottleneck of the transition from the multi-range inhibition of vessels to the fine remodeling of vascular ecology. These prospective insights provide a theoretical basis and practical ideas for strengthening type H vessel interventions in the treatment of KOA.

## Figures and Tables

**Table 1:** **Functional characterization of HIF, SLIT, DLL4/Notch, HIF/mTOR, HIF/SLIT, and LOX-1/HIF-1α/SLC7A11 in OA.**

Model	Signal Pathway Category	Upstream Target	Signal Axis	Downstream Related Targets	Function	References
Mouse model induced by papain solution	HIF-1α/VEGF	?	HIF-1α/VEGFα	HIF-1α↓, VEGFα↓ CD31^hi^EMCN^hi^ type H endothelial cells in subchondral bone↓	Type H endothelial cells in subchondral bone↓	[40]
DMM mouse model	HIF-1α/VEGF	PHD2↑	PHD2/HIF-1α/VEGFα	HIF-1α↓, VEGFα↓ CD31^+^EMCN^+^ type H endothelial cells in subchondral bone↓	Type H endothelial cells in subchondral bone↓	[41]
OA cell model, OA rat model	HIF-1α/VEGF	RAD54↑	RAD54L/HIF-1α/VEGF	TNF-α↓, IL-6↓, IL-18↓ HIF-1α↓, VEGF↓	HIF-1α/VEGF signaling pathway in chondrocytes↓	[42]
ACLT mouse model	HIF-1α/VEGF	Lcp1↓	Lcp1/subchondral osteoclasts/Type H vessels/HIF-1α	COL II↑, aggrecan↑ MMP13↓, ADAMTS5↓, COL X↓ CD31^hi^EMCN^hi^ type H vessels↓	Type H vessels in subchondral bone↓	[43]
Rat model induced by sodium iodoacetate	HIF-2α/VEGF	NF-κB↓	NF-κB/HIF-2α	Rela(NF-κB p65 subunit)↓, Epas1↓ TNF-α↓, IL-6↓, IL-1β↓ Mmp13↓	Inflammatory response factors in chondrocytes↓, Epas1 in chondrocytes↓, collagen degrading enzymes↓	[44]
Chondrocyte model, Early OA mouse model	HIF-2α/VEGF	IHH↓	IHH/GLI-1/HIF-2α	GLI-1↓, HIF-2α↓, VEGF↓ COL X↓	Abnormal angiogenesis in subchondral bone↓	[45]
Primary rat condylar chondrocyte model induced by IL-1β, The TMJ-OA rat model was induced by unilateral anterior tooth reversal	HIF-2α/VEGF	?	HIF-2α/VEGF	HIF-2α↓, MMP13↓, VEGF↓	HIF-2α in chondrocytes↓, MMP13 in chondrocytes↓, VEGF in chondrocytes↓	[46]
Comparison of knee joint cartilage tissue between OA patients and normal person, Culture of primary human OA chondrocyte in vitro	HIF-3α/VEGF	miR-210↑	miR-210/HIF-3α	HIF-3α↓ COL2A1↑ COL10A1↓, MMP13↓	Cartilage hypertrophy markers COL10A1 and MMP13↓	[47]
Multi-directional differentiation functional model of rat bone marrow mesenchymal stem cell	HIF-3α/VEGF	miR-210-3p↑	miR-210-3p/HIF-3α	HIF-3α↓ SOX9↑, COL II↑ peroxisome proliferator-activated receptor gamma↓, lipoprotein lipase↓	the chondrogenesis ability of rat bone marrow mesenchymal stem cells ↑, inhibit their differentiation into adipocytes	[48]
lumbar facet joint OA model	HIF-3α/VEGF	miR-210-3p↑	miR-210-3p/HIF-3α	HIF-3α↓ MMP-13↓ p16↓, p21↓, p53↓	chondrogenesis and anti-aging ability↑	[49]
ACLT mouse model	SLIT3	?	SLIT3/ROBO1/Type H vessels	SLIT3↓ CD31↓, EMCN↓	Number of CD31^+^EMCN^+^ type H endothelial cells in subchondral bone↓	[50]
Temporomandibular joint osteoarthritis model	SLIT3	?	Subchondral Bone Osteoclasts/SLIT3/Abnormal Sensory Nerve	TRAP^+^osteoclast↓ SLIT3↓ Abnormal sensory nerve↓	Abnormal nerves in subchondral bone↓	[51]
Temporomandibular joint osteoarthritis mouse model, Rat primary condylar chondrocyte model	SLIT3	Plexin-B2↑	Plexin-B2/YAP/SLIT3/Type H vessels	Plexin-B2↑, YAP↑ SLIT3↑ Type H angiogenesis↑	Subchondral type H angiogenesis↑	[52]
Human OA cartilage, OA cell model	HIF/mTOR	LncHIFCAR↓	PI3K/AKT/mTOR HIF-1α/VEGF	PI3K↓, AKT↓, mTOR↓ HIF-1α↓, VEGF↓	chondrocyte apoptosis and inflammatory response↓	[53]
OA mouse model, IL-1β-induced inflammation-iron death model in primary chondrocytes	HIF/mTOR	AMPK↑	AMPK/mTOR/HIF-1α	mTOR↓, HIF-1α↓ Malondialdehyde↓, Fe^2+^↓ GSH↑, GPX4↑, ferritin↑ prostaglandin E2↓, nitric oxide↓ MMP1↓, MMP3↓	Pathological type H vessels invading subchondral bone↓	[54]
Von Hippel–Lindau cartilage specific knockout mice model, DMM mouse model	HIF/mTOR	Von Hippel–Lindau↓	HIF-2α/mTOR	HIF-2α↑, mTOR↑,	Chondrocyte apoptosis↑, the progression of age-related and DMM-induced OA↑	[55]

↑ Upregulation or Promotion; ↓ Downregulation or Inhibition. Abbreviations: ACLT, anterior cruciate ligament transection; ADAMTS5, a disintegrin and metalloproteinase with thrombospondin motifs 5; AKT, AKT serine/threonine kinase; AMPK, adenosine monophosphate-activated protein kinase; CD31, platelet endothelial cell adhesion molecule-1; COL II, collagen type II; COL X, collagen type X; COL2A1, collagen type II alpha 1 chain; COL10A1, collagen type X alpha 1 chain; DMM, destabilization of the medial meniscus; EMCN, endomucin; Epas1, endothelial PAS domain protein 1; EPC, endothelial progenitor cell; FGF2, fibroblast growth factor 2; FGFR2, fibroblast growth factor receptor 2; GLI-1, glioma-associated oncogene homolog-1; GPX4, glutathione peroxidase 4; GSH, glutathione; HIF-1α, hypoxia-inducible factor 1-alpha; HIF-2α, hypoxia-inducible factor 2-alpha; HIF-3α, hypoxia-inducible factor 3-alpha; HUVEC, human umbilical vein endothelial cell; IHH, Indian hedgehog; IL-1β, interleukin-1 beta; IL-6, interleukin-6; IL-18, interleukin-18; Lcp1, lymphocyte cytosolic protein 1; LncHIFCAR, long noncoding HIF-1α co-activating RNA; miR, microRNA; MMP, matrix metalloproteinase; mTOR, mechanistic target of rapamycin; NF-κB, nuclear factor kappa B; OA, osteoarthritis; OARSI, Osteoarthritis Research Society International; OPN, osteopontin; OVX, ovariectomized; p16, cyclin-dependent kinase inhibitor 2A; p21, cyclin-dependent kinase inhibitor 1A; p53, tumor protein p53; PDGFR-β, platelet-derived growth factor receptor beta; PHD2, prolyl hydroxylase domain-containing protein 2; PI3K, phosphoinositide 3-kinase; RAD54L, RAD54-like; Rela, RELA proto-oncogene, NF-κB p65 subunit; ROBO1, roundabout guidance receptor 1; SLIT, slit guidance ligand; SOX9, SRY-box transcription factor 9; TGF-β1, transforming growth factor beta 1; THVEC, type H vascular endothelial cell; TMJ-OA, temporomandibular joint osteoarthritis; TNF-α, tumor necrosis factor-alpha; TRAP, tartrate-resistant acid phosphatase; TRIM65, tripartite motif-containing protein 65; VEGF, vascular endothelial growth factor; YAP, Yes-associated protein.

**Table 2:** **Pharmacological modulation of type H endothelial cell homeostasis in KOA.**

Classification	Treatment	Experimental Model	Mechanism	Effect	References
Small molecule signaling modulators	Angptl7 knockdown; Alofanib (FGFR2 inhibitor)	KOA mouse model; HUVEC co-culture model	Blocks FGF2/FGFR2 axis; preserves cartilage matrix. Inhibits FGF2/FGFR2 signaling; reduces HUVEC tube formation.	Inhibits abnormal subchondral type H endothelial cell formation	[8]
	AAV9 injection (anti-PDGFR-β)	ACLT-induced OA mouse model	Inhibits talin1/FAK signaling via PDGFR-β mediation.	Blocks abnormal subchondral type H endothelial cell proliferation.	[9]
	SLIT3 knockdown	HUVEC tube formation model	Inhibits Robo1/TGF-β1/SMAD pathway; reduces VEGF/CD31/EMCN expression.	Reduces OARSI scores and the number of CD31^+^EMCN^+^ type H endothelial cells in OA.	[50]
	Silent LOX-1	High glucose-treated rat THVEC model	Activation of HIF-1α/SLC7A11/GPX4 signaling axis, inhibits ferroptosis	Promote the generation of type H endothelial cells	[107]
	Stattic (Stat3 inhibitor)	DMM-induced OA mouse model	Downregulates VEGF, Angiopoietin-2, and IL-6.	Inhibits subchondral type H endothelial cell generation and inflammation.	[111]
	Osteoclast-specific EP4 knockout; Novel EP4 antagonist	OA patient subchondral bone; osteoclast-specific EP4 knockout (EP4^LysM^) OA mouse model; OA mouse model; in vitro cell experiments	Downregulates PDGF-BB, sensory nerve innervation, and aberrant subchondral type H vessel proliferation	Inhibits aberrant subchondral type H vessel proliferation	[112]
	LY294002(PI3K/AKT signal axis inhibitor)	ACLT + DMM-induced OA mouse model	Inhibits PI3K/AKT/OPN axis.	Reduces cartilage damage; suppresses MMP13 and TRAP^+^ cells.	[113]
	Enhanced PDGFR-β	Osteoporosis patients and OVX mouse model	Activates PDGFR-β/PAK1/Notch1 axis.	Induces type H phenotype differentiation; promotes vascularized bone regeneration.	[114]
	Knockout RKIP	OVX-induced osteoporosis mouse model	Stabilizes HIF-1α; promotes angiogenic macrophage polarization.	Increases CD31^hi^EMCN^hi^ type H endothelial cells ratio; enhances bone formation.	[115]
					
Biomaterial-mediated reconstruction	3D-printed PCL-SE stent	OA patient subchondral bone tissue; ACLT-induced OA mouse model; EPC in vitro model	Activates Notch and HIF-1/VEGFα axes.	Promotes EPC differentiation into type H endothelial cells; enhances osteogenesis.	[116]
	SNS@DBM bone scaffold	Osteoporotic femoral condyle bone defect rat model	Induces M2 macrophage polarization.	Enhances the generation of type H endothelial cells and bone formation; clears tumors.	[117]
	3D print PTMN holder	Rat calvarial defect model	Activates HIF-1α/STAT3/VEGF and PI3K/AKT pathways.	Improves repair efficiency; increases type H endothelial cells proportion.	[118]
	3D-printed DFO-Au@GN hydrogel scaffold	Rat bone defect model; BMSC/HUVEC co-culture model	Promotes endothelial H-vessel formation.	Enhances osteogenic differentiation of BMSCs.	[119]
	BMP-2/SCS/CPC composite scaffold	Rat calvarial defect model; BMSC/HUVEC co-culture model	Restores skeletal stem cell differentiation.	Promotes the generation of type H endothelial cells and new bone formation.	[120]
					
Natural products	Daidzein	Aged mouse spinal fusion model	Downregulates Caveolin-1; activates EGFR/AKT/PI3K axis.	Enhances BMEC migration; promotes the generation of type H endothelial cells.	[17]
	Dibutyl phthalate	OA rat model	Inhibits osteoclast overactivation and sensory nerve invasion.	Inhibits the generation of subchondral type H endothelial cells.	[38]
	YSTG	OVX-induced osteoporosis mouse model	Upregulates SLIT3 expression.	Promotes the generation of type H endothelial cells in diaphysis.	[88]
	Soybean Isoflavone	Osteoporosis mouse model	Activates TSC1; inhibits mTORC1/VEGF axis.	Reduces the generation of pathological subchondral endothelial cells in OA.	[121]
	SCT-HA hydrogel microspheres	ACLT-induced OA mouse model	Inhibits CD31^+^EMCN^+^ vessels and osteoclasts.	Suppresses pathological angiogenesis in subchondral bone.	[122]
	Polyphenolic tannic acid combined with LL-37	In vitro cell co-culture; in vivo bone defect model	Induces M2 macrophage polarization	Promotes type H angiogenesis and bone regeneration	[123]
					
Anti-aging drugs	ABT263 (Navitoclax)	ACLT-induced OA mouse model	Clears senescent osteoclasts (SnOC).	Inhibits the generation of type H endothelial cells in the endplate. ; alleviates spinal pain.	[19]
	TRPM7 Knockout	Lumbar instability and aging mouse models	Upregulates p21; downregulates angiogenic genes.	Reduces microvascular density; inhibition of p21 reverses senescence.	[124]
	6-Hydroxydopamine	Endothelial-specific TRPM7 knockout mice; hindlimb ischemia model	Inhibits sympathetic nerves; downregulates calpain 6.	Promotes type H endothelial cells formation and increases osteogenic factor-secreting senescent macrophages.	[125]

Abbreviations: AAV9, adeno-associated virus serotype 9; ACLT, anterior cruciate ligament transection; AKT, AKT serine/threonine kinase; BMEC, bone marrow endothelial cell; BMP-2, bone morphogenetic protein-2; BMSC, bone marrow mesenchymal stem cell; CD31, platelet endothelial cell adhesion molecule-1; CPC, calcium phosphate cement; DFO, deferoxamine; DMM, destabilization of the medial meniscus; EGFR, epidermal growth factor receptor; EMCN, endomucin; EP4, E-prostanoid 4 receptor; EPC, endothelial progenitor cell; FAK, focal adhesion kinase; FGF2, fibroblast growth factor 2; FGFR2, fibroblast growth factor receptor 2; GPX4, glutathione peroxidase 4; HIF-1α, hypoxia-inducible factor 1-alpha; HUVEC, human umbilical vein endothelial cell; IL-6, interleukin-6; KOA, knee osteoarthritis; LL-37, cathelicidin LL-37; LOX-1, lectin-like oxidized low-density lipoprotein receptor-1; MMP13, matrix metalloproteinase 13; mTORC1, mechanistic target of rapamycin complex 1; OARSI, Osteoarthritis Research Society International; OPN, osteopontin; OVX, ovariectomized; p21, cyclin-dependent kinase inhibitor 1A; PAK1, p21-activated kinase 1; PCL-SE, polycaprolactone scaffold modified with EPLQLKM and SVVYGLR peptides; PDGFR-β, platelet-derived growth factor receptor beta; PI3K, phosphoinositide 3-kinase; PTMN, poly(trimethylene carbonate-co-caprolactone)-b-poly(ethylene glycol) nanoparticles; RKIP, Raf kinase inhibitor protein; SCT-HA, selenium-doped carbon quantum dot modified hyaluronic acid hydrogel microspheres; SCS, sulfated chitosan; SLC7A11, solute carrier family 7 member 11; SNS@DBM, siliconene nanosheet-modified demineralized bone matrix; STAT3, signal transducer and activator of transcription 3; TGF-β1, transforming growth factor beta 1; THVEC, type H vascular endothelial cell; TRAP, tartrate-resistant acid phosphatase; TRPM7, transient receptor potential melastatin 7; TSC1, tuberous sclerosis complex 1; VEGF, vascular endothelial growth factor; YSTG, Yi Shen Tiao Gan decoction.

## Data Availability

No new data were created or analyzed in this study. Data sharing is not applicable to this article.

## Supplementary Materials

The following supporting information can be downloaded at: https://www.mdpi.com/article/10.3390/cells15141312/s1. Table S1. Functional characterization of HIF, SLIT, DLL4/Notch, HIF/mTOR, HIF/SLIT, and LOX-1/HIF-1α/SLC7A11 in non-OA disease models.

## Abbreviations

**Abbreviation**	**Full Name**
AAV9	adeno-associated virus serotype 9
ABT263	navitoclax
ACAN	aggrecan
ACLT	anterior cruciate ligament transection
ADAMTS5	a disintegrin and metalloproteinase with thrombospondin motifs 5
ADSC	adipose-derived mesenchymal stem cell
AKT	AKT serine/threonine kinase
AMPK	adenosine monophosphate-activated protein kinase
AngC-2	angiogenic chondrocyte-2
ARF6	ADP-ribosylation factor 6
AuNPs	gold nanoparticles
BMEC	bone marrow endothelial cell
BMP-2	bone morphogenetic protein-2
BMSC	bone marrow mesenchymal stem cell
Bu-Sui-Dan	Bu-Sui-Dan (traditional Chinese medicine formulation)
CALCRL	calcitonin receptor-like receptor
CD31	platelet endothelial cell adhesion molecule-1
CD90	cluster of differentiation 90
CD146	cluster of differentiation 146
COL I	collagen type I
COL II	collagen type II
COL X	collagen type X
COL2A1	collagen type II alpha 1 chain
COL10A1	collagen type X alpha 1 chain
CPC	calcium phosphate cement
CRISPR-Cas9	clustered regularly interspaced short palindromic repeats–CRISPR-associated protein 9
CXCL10	C-X-C motif chemokine ligand 10
DBM	demineralized bone matrix
DFO	deferoxamine
DLL4	delta-like canonical Notch ligand 4
DMM	destabilization of the medial meniscus
EGFR	epidermal growth factor receptor
EMCN	endomucin
eNOS	endothelial nitric oxide synthase
Epas1	endothelial PAS domain protein 1
EP4	E-prostanoid 4 receptor
EPC	endothelial progenitor cell
FAK	focal adhesion kinase
FGF2	fibroblast growth factor 2
FGFR2	fibroblast growth factor receptor 2
FOXO1	forkhead box O1
GelMA	gelatin methacryloyl
GLI-1	glioma-associated oncogene homolog-1
GPX4	glutathione peroxidase 4
GRK2	G protein-coupled receptor kinase 2
GSH	glutathione
HIF-1α	hypoxia-inducible factor 1-alpha
HIF-2α	hypoxia-inducible factor 2-alpha
HIF-3α	hypoxia-inducible factor 3-alpha
HSP90	heat shock protein 90
HUVEC	human umbilical vein endothelial cell
IHH	Indian hedgehog
IL-1β	interleukin-1 beta
IL-2	interleukin-2
IL-6	interleukin-6
IL-18	interleukin-18
KOA	knee osteoarthritis
Lcp1	lymphocyte cytosolic protein 1
LL-37	cathelicidin LL-37
LncHIFCAR	long noncoding HIF-1α co-activating RNA
LOX-1	lectin-like oxidized low-density lipoprotein receptor-1
M1	classically activated macrophage
MAPK	mitogen-activated protein kinase
miR	microRNA
miR-29a-3p	microRNA-29a-3p
miR-29cb2	microRNA-29cb2
miR-148b-3p	microRNA-148b-3p
MMP	matrix metalloproteinase
mTOR	mechanistic target of rapamycin
mTORC1	mechanistic target of rapamycin complex 1
MXene (Nb_2_C)	niobium carbide MXene
NAPVSIPQ	Asn-Ala-Pro-Val-Ser-Ile-Pro-Gln
NF-κB	nuclear factor kappa B
NIPAM	N-isopropylacrylamide
Notch	Notch receptor
OA	osteoarthritis
OARSI	Osteoarthritis Research Society International
OCN	osteocalcin
OPN	osteopontin
Osx	Osterix
OVX	ovariectomized
p16	cyclin-dependent kinase inhibitor 2A
p21	cyclin-dependent kinase inhibitor 1A
p53	tumor protein p53
PAK1	p21-activated kinase 1
PCL-SE	polycaprolactone scaffold modified with EPLQLKM and SVVYGLR peptides
PDGF-BB	platelet-derived growth factor BB
PDGFR-β	platelet-derived growth factor receptor beta
PHD2	prolyl hydroxylase domain-containing protein 2
PI3K	phosphoinositide 3-kinase
Plexin-B2	plexin B2
PTMN	poly(trimethylene carbonate-co-caprolactone)-b-poly(ethylene glycol) nanoparticles
RAD54L	RAD54-like
Rela	RELA proto-oncogene, NF-κB p65 subunit
RKIP	Raf kinase inhibitor protein
ROBO1	roundabout guidance receptor 1
ROS	reactive oxygen species
RUNX2	runt-related transcription factor 2
SCS	sulfated chitosan
SCT-HA	selenium-doped carbon quantum dot modified hyaluronic acid hydrogel microspheres
SDF-1α	stromal cell-derived factor 1α
SHH	sonic hedgehog
SIRT1	sirtuin 1
SLC7A11	solute carrier family 7 member 11
SLIT	slit guidance ligand
SLIT2	slit guidance ligand 2
SLIT3	slit guidance ligand 3
SnOC	senescent osteoclast
SNS	silicon nanosheets
SNS@DBM	siliconene nanosheet-modified demineralized bone matrix
SOX9	SRY-box transcription factor 9
STAT3	signal transducer and activator of transcription 3
TGF-β1	transforming growth factor beta 1
THVEC	type H vascular endothelial cell
TMJ-OA	temporomandibular joint osteoarthritis
TNF-α	tumor necrosis factor-alpha
TRAP	tartrate-resistant acid phosphatase
TRIM65	tripartite motif-containing protein 65
TRPM7	transient receptor potential melastatin 7
TSC1	tuberous sclerosis complex 1
VEGF	vascular endothelial growth factor
VEGFα	vascular endothelial growth factor A
YAP	Yes-associated protein
YSTG	Yi Shen Tiao Gan decoction
ZBTB16	zinc finger and BTB domain-containing protein 16
αSMA	alpha-smooth muscle actin
β-TCP	beta-tricalcium phosphate
