# Supplementary material for: Pharmacological Targeting of Type H Endothelial Cells in Knee Osteoarthritis: From Molecular Signaling to Cellular Homeostasis
Source: Cells. 2026 Jul 22;15(14):1312. doi: 10.3390/cells15141312 (PMC13407103; doi:10.3390/cells15141312)
Supplement: Supplementary file 1 [file cells-15-01312-s001.zip › cells-4398209-supplementary.pdf]

Table S1. Functional characterization of HIF, SLIT, DLL4/Notch, HIF/mTOR, HIF/SLIT, and LOX-1/HIF-1α/SLC7A11 in non-OA disease models.

Model	Signal pathway category	Upstream target	Signal axis	Downstream related targets	Function	References
MA condylar osteogenesis mouse model	DLL4/Notch	?	DLL4/Notch/Type H vessels	DLL4/Notch↓<br>Type H vessels↓<br>RUNX2^{+} bone progenitor cells↓, Noggin↓	The number of type H vessels in the mandible↓	[33]
Unilateral nasal obstruction mouse model,<br>Unilateral nasal obstruction + MA mouse model	HIF-1α/VEGF	?	Hypoxia/HIF-1α/Type H vessels in subchondral bone	HIF-1α↑, Type H vessels↑, Osx^{+} cells in subchondral bone ↑	Type H vessels in subchondral bone ↑	[54,55]
Pulmonary hypertension related model	HIF-2α/VEGF	ARF6↓	ARF6/HIF-2α	HIF-2α↓<br>Angiogenesis↓	Human pulmonary artery endothelial cells↓	[65]
OVX mouse model	HIF-3α/VEGF	miR-29cb2↓	miR-29cb2/HIF-3α	HIF-3α↑<br>HIF-1α↓, VEGF↓<br>CD31^{hi}EMCN^{hi} type H endothelial cells↓	CD31^{hi}EMCN^{hi} type H endothelial cells in the femur↓	[74]
High glucose human retinal endothelial cell model,<br>Oxygen-induced retinopathy mouse model	HIF-3α/VEGF	TRIM65↑	TRIM65/miR-29a-3p/HIF-3α	miR-29a-3p↓<br>HIF-3α↑, VEGFα↑<br>Pathological neovascularization↑	Pathological retinal neovascularization↑	[76]
Rat model of diabetic retinopathy	HIF-3α/VEGF	?	HIF-3α/HIF-1α/VEGF<br>HIF-3α/HIF-2α/VEGF	HIF-3α↑<br>HIF-1α↓, HIF-2α↓, VEGF↓	Pathological retinal neovascularizations↓	[77]
A mouse model of tendon–bone healing	SLIT3	CALCRL↑	CALCRL/SLIT3/Type H vessels	SLIT3↑<br>CD31^{hi}EMCN^{hi} type H endothelial cells↑<br>SHH↑	CD31^{hi}EMCN^{hi} type H endothelial cells in the tendon–bone interface↑, and positively regulate sensory nerves, which is conducive to bone regeneration and functional recovery	[84]
Aromatase inhibitor relate bone loss model	SLIT3	?	SLIT3/Type H vessels in bone	SLIT3↑<br>CD31↑, EMCN↑, Osx↑	The number of type H endothelial cells in bone↑	[85]
OVX mouse model,<br>GPX4 knockout mouse model,<br>BMSC-HUVEC coculture,<br>Iron dead cell model	SLIT3	GPX4↑	GPX4/BMSC Iron Death/SLIT3/ROBO1/Type H vessels	GPX4↑<br>SLIT3↑<br>CD146^{+} bone progenitor cells↑,<br>VEGFα↑, CD31^{+}EMCN^{+} type H endothelial cells↑	CD31^{+}EMCN^{+} type H endothelial cells in the tibia↑	[86]
Rib injury model in CD90 knockout mice,<br>In vitro cell co-culture model	DLL4/Notch	CD90↓	CD90/DLL4/Notch/<br>Type H vessels	DLL4/Notch↓<br>Number of CD31^{+}EMCN^{+} type H endothelial cells↓	CD31^{+}EMCN^{+} type H endothelial cell number and vessel volume in the ribs↓, Addition of recombinant DLL4 protein or overexpression of Notch1	[91]

					reversed the above responses	
Natural aging osteoporosis mouse model	DLL4/Notch	Bu-Sui-Dan	Zinc finger E-box-binding homeobox 1/DLL4/Notch1	Zinc finger E-box-binding homeobox 1↑, DLL4↑, Notch1↑,	Type H vessel formation in the femur↑, improve bone mineral density and trabecular bone microstructure	[92]
Alveolar bone defect model, ADSC-HUVEC co-culture angiogenesis model	HIF/mTOR	?	HIF-1α/VEGF/AKT/mTOR	HIF-1α↑, VEGF↑, AKT↑, mTOR↑ Osteogenic differentiation capacity↑ Endothelial cell tube-forming ability↑	Endothelial cell tube-forming ability in alveolar bone↑	[97,98]
Rabbit model of intervertebral disk degeneration	HIF/mTOR	AKT↑	AKT/mTOR/HIF-1	AKT↑, mTOR↑, HIF-1↑	Inhibition of inflammatory response and apoptosis in the intervertebral disk, thereby delaying degeneration	[100]
Model of alveolar bone disuse osteoporosis	HIF/SLIT	?	HIF-1α/SLIT3/Type H vessels	HIF-1α↓ SLIT3↓ Type H endothelial cells↓ Osx^{+} osteoblastic progenitor cells↓	Type H endothelial cells in alveolar bone↓	[102]
Vascular graft restenosis model	HIF/SLIT	?	HIF-1α/SLIT2	HIF-1α↑ SLIT2↓ Migration and proliferation of vascular smooth muscle cell↑	Migration and proliferation of vascular smooth muscle cell↑	[103]
High glucose-induced iron death in rat H-type vascular endothelial cells	LOX-1/HIF-1α/ SLC7A11	LOX-1↓	LOX-1/HIF-1α/ SLC7A11/GPX4	HIF-1α↑ SLC7A11↑ GPX4↑	Inhibition of ferroptosis of THVEC induced by hyperglycemia, thereby restoring the integrity and function of type H vessels	[107]

↑ Upregulation or Promotion ; ↓ Downregulation or Inhibition

Abbreviations: ADSC, adipose-derived mesenchymal stem cell; AKT, AKT serine/threonine kinase; ARF6, ADP-ribosylation factor 6; BMSC, bone marrow mesenchymal stem cell; Bu-Sui-Dan, Bu-Sui-Dan (traditional Chinese medicine formulation); CALCRL, calcitonin receptor-like receptor; CD146, cluster of differentiation 146; CD31, platelet endothelial cell adhesion molecule-1; CD90, cluster of differentiation 90; DMM, destabilization of the medial meniscus; DLL4, delta like canonical Notch ligand 4; EMCN, endomucin; GPX4, glutathione peroxidase 4; HIF-1α, hypoxia-inducible factor 1-alpha; HIF-2α, hypoxia-inducible factor 2-alpha; HIF-3α, hypoxia-inducible factor 3-alpha; HUVEC, human umbilical vein endothelial cell; LOX-1, lectin-like oxidized low-density lipoprotein receptor-1; MA, mandibular advancement; miR, microRNA; mTOR, mechanistic target of rapamycin; OA, osteoarthritis; Osx, Osterix; OVX, ovariectomized; PI3K, phosphoinositide 3-kinase; ROBO1, roundabout guidance receptor 1; RUNX2, runt-related transcription factor 2; SHH, sonic hedgehog; SLC7A11, solute carrier family 7 member 11; SLIT, slit guidance ligand; THVEC, type H vascular endothelial cell; TRIM65, tripartite motif-containing protein 65; VEGF, vascular endothelial growth factor; YAP, Yes-associated protein.
